# Measurement of vinyl acetate monomer in consumer products and modeled estimates of consumer exposure

**DOI:** 10.1038/s41370-025-00786-y

**Published:** 2025-06-18

**Authors:** Alison Gauthier, William Behymer, Jennifer Bare, Mandie Kramer, Wade T. Barranco, Joseph P. Longtin, Susan Borghoff, Andrew Jaques

**Affiliations:** 1 ToxStrategies, LLC, Katy, TX USA; 2 Lyondell Chemical Company, Houston, TX USA; 3 ToxStrategies, LLC, Asheville, NC USA; 4 Vinyl Acetate Council, Washington, DC USA

**Keywords:** Vinyl acetate, Consumer product, Exposure

## Abstract

**Background:**

Vinyl acetate monomer (VAM) (CAS 108-05-4) is employed in the creation of an array of polymers and copolymers used in the manufacture of consumer products. There is no direct use of VAM in consumer products. However, residual amounts of unreacted VAM in (co)polymer products have been identified as a possible general population exposure concern.

**Objective:**

The objective of this evaluation was to provide a contemporary review of exposure to VAM via residual VAM (co)polymer in a range of consumer products in the United States.

**Methods:**

The study authors conducted a market-basket sampling of residual VAM levels in 71 consumer products purchased in the United States that met the selection criteria. Subsequently, exposure assessments were conducted using ConsExpo (version 1.1.1) and the United States Environmental Protection Agency’s Consumer Exposure Model (CEM; version 2.1) on a subset of those identified products.

**Results:**

Of the products analyzed, 40 had VAM concentrations below the lowest detection limits (0.1–2 ppmw), 19 were non-detectable but the product materials demonstrated atypical or nonlinear calibration behavior, four had detectable VAM in the 2–10 ppmw range, and eight had detectable VAM from 10 to 648 ppmw. Eleven use scenarios were developed based on seven categories of consumer products from the evaluation. Resulting exposure estimates were all less than both acute and chronic non-cancer human health thresholds.

**Impact:**

- This study presents an analytical detection methodology for residual VAM present in a variety of consumer products. The information presented herein can inform future studies of VAM exposure and facilitate exposure estimates of VAM in similar products by providing measured concentrations. This study also demonstrates that, for the products evaluated, potential VAM exposure is less than acute and chronic health thresholds for the general public based on modeled exposure estimates.

## Introduction

Vinyl acetate monomer (VAM), also known as acetic acid ethenyl ester (CAS 108-05-4), is used in the creation of an array of polymers and copolymers. Polyvinyl acetate (PVA) is formed by aqueous free-radical emulsion polymerization whereby water, a surfactant, and an initiator are mixed, heated, and combined with VAM (homopolymer), whereas copolymers are made from the reaction of VAM and other monomers. Since VAM readily polymerizes with co-monomers, it can be used to create a wide range of polymers that achieve different and unique final product properties. These VAM-based (co)polymers have numerous applications, including in coatings, adhesives, personal care products, films, and a range of consumer products.

VAM exhibits low acute toxicity as evidenced by studies in animals (rat oral LD50 = 3500 mg/kg; rabbit dermal LD50 = 7440 mg/kg; rat inhalation LC50 = 14,084 mg/m^3^ (4000 ppm) [4 h]), while repeated dosing elicits respiratory tract irritation [1]. Mild skin and eye irritation has been noted in rabbits and genotoxicity has been noted in vitro in mammalian cells (clastogenicity) [1]. VAM is considered a point-of-contact animal carcinogen as supported by nasal tumors identified in rats following inhalation and gastrointestinal tract tumors noted in rodents following oral intake [1].

There is no direct use of VAM in consumer products, however, residual amounts of unreacted VAM in (co)polymer products have been identified as a possible general population exposure concern. Governmental assessments in Canada [1] and the European Union [2, 3] have determined that the levels of residual VAM in consumer products are low and do not create exposures of concern. There has not been an assessment of VAM in consumer products in the United States.

The acquisition of measured VAM concentrations in consumer polymer products was a key component in the 2008 Canadian exposure and risk assessment [1]. The Celanese Corporation sampled 136 vinyl acetate polymer products from Canada, obtained directly from retail stores, to develop a database of VAM residual levels in consumer products. This research found that 74% (101 of the 136 samples) of the products tested had residual monomer content of <25 ppm (0.0025%), the analytical limit of detection (LOD) of VAM by product weight. Of the 26% of products that had detectable VAM, adhesives and glues generally had the highest levels of residual concentrations. Most products were in the range of 25–300 ppm, with the highest sample at just over 2000 ppm (0.2%). These testing results were submitted to the Canadian government in July 2008 and used by Health Canada to revise and improve its exposure and risk assessments for VAM. Health Canada concluded, based in part on these data, that VAM, “is not currently entering the environment in a quantity or concentration or under conditions that constitute or may constitute a danger in Canada to human life or health” [1].

The objective of this evaluation is to provide a contemporary review of exposure to VAM via VAM (co)polymer consumer products in the United States. This project included the collection of consumer product samples purchased from various retail outlets in the United States, followed by their analysis for residual levels of VAM. Subsequently, exposure assessments were conducted on a subset of those identified products to estimate potential human exposure and characterize potential health risk using ConsExpo (version 1.1.1) and the U.S. Environmental Protection Agency’s (USEPA) Consumer Exposure Model (CEM; version 2.1). ConsExpo is a web-based exposure modeling tool maintained by the Dutch National Institute for Public Health and the Environment (RIVM) that contains built-in exposure models covering a multitude of consumer products such as cosmetics and do-it-yourself products [4]. USEPA’s Consumer Exposure Model includes 21 models used to estimate emissions and exposures to products or articles, including paints and plastic articles [5]. Exposure model selection per product group was based on using the appropriate model for the product application and exposure scenario as described in the “Materials and methods” section.

## Materials and methods

### Assessment approach

The VAM exposure and risk assessment involved the identification and acquisition of VAM-based (co)polymer consumer products, chemical analysis of products for residual VAM, and development of exposure scenarios and computer modeling to estimate plausible exposure concentrations. The estimated exposures were then compared to available health benchmarks.

### Consumer product selection process

Identification of consumer products potentially containing residual vinyl acetate was conducted via two resources. One source was the Consumer Product Information Database (CPID; https://www.whatsinproducts.com/), whereby product lists were obtained using the search terms: “ethylene vinyl acetate,” “ethylvinyl acetate,” “PVA,” “polyvinyl alcohol,” and “vinyl acetate.” In an effort to replicate consumer product purchasing habits, the second source used to identify consumer products was Amazon.com. The same CPID database search terms were used to query Amazon.com for relevant products. Verification of the inclusion of vinyl acetate or vinyl acetate co-polymers in products was based on statements on the Amazon.com product webpage, retrieval of manufacturer-generated technical data sheets, or both when possible. Food and food packaging products were excluded from this analysis because CPID does not include packaged foods, and no food or food packaging products were identified through Amazon.

Exclusion of some products from ordering/testing included comparable products already included in the final testing list, lack of availability for purchase (e.g., discontinued or out-of-stock), cost-prohibitive shipping fees (e.g., bulk products), inability to identify which sub-part of the product contained the chemical of interest, vendor repeatedly shipping the wrong product, or incompatibility with analytical testing method (e.g., nail polish).

A total of 71 products were included in the chemical analysis. All products were shipped directly from the distributor to the analytical laboratory for testing and were disposed of appropriately following testing.

### Sample preparation for analysis of residual VAM

Powders, pastes, and liquid products were introduced directly into headspace gas chromatograph vials without any additional preparation. Molded articles and other rigid materials were cut into small pieces or strips, both to facilitate introduction into the headspace vial as well as to minimize the VAM equilibration time. Pieces cut from thicker molded and rigid articles were typically ¼-in. cubes, while strips cut from thin sheet and film articles were typically 1–2 in. long by ¼–½ in. wide. Samples were prepared for analysis by introducing the targeted weights or volumes into 20 mL (nominal) glass headspace vials, sealing the vials with aluminum crimp caps and Teflon-lined butyl rubber septa. The typical sample target weight for solid samples was 1.00 g and the target for liquid samples added volumetrically was 1.00 mL. For cases in which a particular sample bulk density was exceptionally low (e.g., highly foamed molded article) or a limited amount of sample was available, a target weight less than 1 g was used.

For solid samples prepared by weight, rather than record the individual sample aliquot weights, aliquots were prepared to within a specified precision (typically ±2%) of the target weight and the nominal target weight value was used as the sample aliquot weight in the calculation of spiked sample concentrations. For liquid samples added using a volumetric pipette, each of several aliquots was weighed and the average of these individual weights was recorded and used as the sample aliquot weight in the calculation of spiked sample concentrations.

### Analytical methods for consumer product analysis

A headspace/gas chromatographic (HS/GC) method was developed and used for the determination of the concentration of residual VAM present in a wide variety of consumer products selected. Samples were sealed in glass headspace vials and heated to 90 °C for 4 h to allow the residual VAM to reach its equilibrium vapor phase concentration. Equilibration studies were conducted on several samples of varying physical properties to ensure VAM equilibrium had been reached. Selected samples were evaluated from 30 to 120 min. The equilibrium time for examined samples was established at 120 min, then doubled to account for potential unforeseen sample differences. Figure S4 illustrates representative equilibration times observed for samples of disparate equilibration kinetics. The headspace was sampled and analyzed for VAM via capillary gas chromatography (GC). The gas chromatograph was equipped with a split/splitless inlet, Dean’s Switch, dual GC columns (primary and secondary), and dual flame ionization detectors (FID). As the VAM peak eluted from the primary column, the Dean’s Switch was activated momentarily to divert the peak to the secondary column, a process referred to as heartcut collection, for additional separation prior to detection. The use of dual FIDs allowed for the simultaneous detection of analytes eluting from both the primary and secondary columns throughout the entire analysis. The calibration for each sample was performed by preparing and analyzing, under the same conditions, additional replicates of the sample, which had been spiked with known quantities of VAM. The residual VAM concentration in the original sample was then determined using the method of standard addition. A chromatogram from this method is provided in the Supplementary Information.

The highest concentration liquid standard was prepared by diluting 2.00 g of VAM, weighed to 0.1 mg, to a total volume of 10.0 mL with acetone. This standard contained 200 mg/mL (or 200 μg/μL) of VAM. Serial dilutions of this standard were subsequently prepared to obtain a complete series of liquid standards at VAM concentrations of 200, 100, 50, 25, 10, and 2 μg/μL. These standards provide concentrations in the range of 200 to 2 parts per million by weight (ppmw) of VAM when 1 μL is added to a 1 g aliquot of sample. Calibration standards consisted of additional replicates of a given sample to each of which was added 1.00 μL of the desired liquid calibration standard.

Samples were initially screened for VAM content by preparing and analyzing one neat sample replicate as well as a second equally sized sample replicate spiked with 1 μL of the 10 μg/μL VAM standard. The VAM content was estimated from this screening analysis, and based on this estimation, an appropriate quantitation sample set was prepared according to the protocols described below.

If VAM was not detected, two neat sample replicates as well as two sample replicates spiked with the 2 μg/μL standard were prepared and analyzed. The average VAM peak area from the two calibration replicates was used to estimate a LOD for the sample. If VAM was detected and estimated at <2 ppmw, five neat sample replicates as well as two sample replicates spiked with the 2 μg/μL standard were prepared and analyzed. A single-point calibration was used to determine the concentration of residual VAM in the sample. The five neat sample replicates were used to calculate the precision of the determination. If VAM was detected and estimated at 2–10 ppmw, five neat sample replicates as well as two spiked sample replicates for each of the 2 and 10 μg/μL standards were prepared and analyzed. The calibration curve resulting from the linear regression of the data from the five sample replicates and two replicates each of the two calibration samples was used to determine the concentration of residual VAM in the sample. The five neat sample replicates were used to calculate the precision of the determination. If VAM was detected and estimated at >10 ppmw, five neat sample replicates as well as 2 spiked sample replicates for each of the 10, 25, 50, 100, and 200 μg/μL standards were prepared and analyzed. The calibration curve resulting from the linear regression of the data from the five sample replicates and two replicates each of the five calibration samples was used to determine the concentration of residual VAM in the sample. The five neat sample replicates were used to calculate the precision of the determination [6]. Example calibration plots and exact instrument conditions can be found in the Supplementary Information.

### Exposure and risk assessment methods

The VAM exposure assessment was conducted for representative products. Each of the 71 products analyzed were grouped into product use categories based on similar uses and exposure scenarios. The product groups included ingestible, cosmetics/personal care, glues, caulks/sealants, surface treatments, paints, and solid articles. Based on availability, only one ingestible product was selected for the exposure assessment, and 10 products from the remaining categories were also selected. The 10 additional products represented those with higher exposure potential and use by both adult and child consumers. Within each category, the product with the highest measured concentration was selected for modeling. When no products in a category had detectable levels of VAM, the product with the highest detection limit (and therefore the highest assumed potential exposure) and the highest exposure potential (based on surface area, contact frequency, exposure duration, or mass of product used) was selected. Two representative products were chosen for a single category when there were products intended for both adult and child use within the same category or multiple models were identified to represent the different products. For instance, all products in the caulks/sealants category were represented by the same model, whereas the surface treatment category included products where exposure could be estimated by multiple wall and floor models. The products modeled included lip gloss, a face mask, arts and crafts glue, seam adhesive, caulk, joint compound, concrete resurfacer, primer, shelf liner, and an electronic tablet cover; the products and their respective product use categories are listed in Table 1.

**Table 1:** Product categorization and justification for selection.

Product category	Product name	Adult or Child	Concentration of VAM	Justification
Ingestibles	Dietary supplement tablet	Adult	ND (<0.1 ppmw)	Only product in category
Cosmetics/personal care	Lip gloss	Child	ND (<0.2 ppmw)	Entire category is ND; children’s product
	Face mask	Adult	ND (<0.2 ppmw)	Entire category is ND; adult product with highest dermal surface area
Glues	Arts and crafts glue	Adult and child	648 ppmw	Highest detected value for category and universal glue model; child use possible
	Seam adhesive	Adult	74 ppmw	Highest detected value for construction glue
Caulks/sealants	Caulk	Adult	7.6 ppmw	Highest detected value for category; all products represented by the same model
Surface treatment	Joint compound	Adult	ND (<200 ppmw)	Entire category is ND; highest ND value in category for wall-applied products
	Concrete resurfacer	Adult	16 ppmw	Highest detected value for floor-applied products
Paint	Primer	Adult	ND (<6 ppmw)	Entire category is ND; highest ND value in category
Solid article	Shelf liner	Adult	ND (<0.1 ppmw)	Entire category is ND; adult product with highest surface area
	Electronic tablet cover	Child	ND (<0.1 ppmw)	Entire category is ND; children’s product with highest contact frequency

*ND* = not detected.

VAM exposure estimates were made using mathematical models including ConsExpo (version 1.1.1) and U.S. Environmental Protection Agency’s (USEPA) Consumer Exposure Model (CEM; version 2.1). ConsExpo is a web-based exposure modeling tool maintained by the Dutch RIVM that contains built-in exposure models covering a multitude of consumer products such as cosmetics and do-it-yourself products [4]. The USEPA’s CEM includes 21 models used to estimate emissions and exposures to products or articles, including paints and plastic articles [5]. Both models require physical-chemical properties of the compound of interest and have a series of default parameters used to characterize exposure scenarios that may be replaced with more refined estimates for the specific exposure scenario modeled. ConsExpo reports acute and chronic air concentrations, external doses, and internal doses [7], while CEM estimates acute and chronic air concentrations and internal doses [5]. The best model selected between ConsExpo and CEM for each product is based on best fit of the exposure scenarios, with the exception of the ingestible, which was estimated via direct ingestion of a single serving size per day. The utility of ConsExpo and CEM has been demonstrated in several consumer product safety assessments or model comparisons, including for air fresheners, paints, and cleaners [8–10]. Table 2 is the conceptual exposure model for all products and shows the model choice and associated model exposure scenarios, application, user, and relevant exposure routes.

**Table 2:** Conceptual exposure model.

Product	Model	Scenario	Application	Exposure route			
				Inhalation	Dermal	Hand-to-mouth	Oral
Dietary supplement tablet	Direct oral intake calculation	Not Applicable	Adult application				X
Lip gloss	ConsExpo	Cosmetics as toys	Child application				X
Face mask	ConsExpo	Face pack	Adult application		X		
Arts and crafts glue	ConsExpo	Universal wood glue	Adult application	X	X		
			Child application	X	X		
Joint compound	ConsExpo	Joint compound	Adult application	X	X		
Caulk	ConsExpo	Joint sealant	Adult application	X	X		
Seam adhesive	ConsExpo	Tube glue	Adult application	X	X		
Concrete resurfacer	ConsExpo	Floor equalizer	Adult mixing and loading	X	X		
			Adult application	X	X		
Primer	CEM	Water-based wall paint	Adult application^a^	X	X		
			Adult post-application^a^	X			
Electronic tablet cover	CEM	Plastic articles: other objects with potential for routine contact	Child application	X	X	X	X
Shelf liner	CEM	Plastic articles: vinyl flooring	Adult application^a^	X	X	X	
			Adult post-application^a^	X	X	X	

^a^One exposure was conservatively estimated assuming that the same adult applied or used the product and experienced the post-application exposure.

To characterize health risk from use of these products, a margin of safety (MOS) or margin of exposure (MOE) was calculated. Both the MOS and MOE are calculated by dividing the point of departure (POD) by the estimated exposure. A MOS was calculated for instances in which the POD was a threshold value established by a regulatory agency and already incorporates safety factors in the value. A MOE was calculated for instances in which an authoritative POD applicable to human exposure was not identified but could be established based on available toxicology data [11, 12]. A target MOS and MOE were 1 and 100, respectively (i.e., products with an MOS or MOE above 1 or 100, respectively, were considered safe).

All PODs are summarized in Table 3. Briefly, peak inhalation exposure concentrations were compared to the USEPA 10-min Acute Exposure Guideline Level (AEGL) [13]. AEGL-1 values are airborne concentrations at which the general population, including susceptible populations, may experience reversible and nondisabling effects (e.g., notable discomfort, irritation). For vinyl acetate, the AEGL-1 values were based on an inhalation study in which humans reported throat irritation [13]. A human interspecies uncertainty factor of 3 was applied to the study’s no-effect level to derive the AEGL-1 value.

**Table 3:** Points of departure (PODs).

Route of exposure	Duration	POD	Uncertainty/ modifying factor	Toxicological threshold	Reference
Inhalation	Peak (10-min)	20 ppm	3	6.7 ppm (24 mg/m^3^)^a^	USEPA AEGL-1 [12]
Inhalation	Acute (1–14 days)	29.1 ppm	30	1 ppm (3.52 mg/m^3^)^b^	ATSDR General Population MRL Respiratory Non-cancer [13]
Inhalation	Chronic (1+ year)	8.52 ppm	30	0.3 ppm (1.06 mg/m^3^)^b^	ATSDR General Population MRL Respiratory Non-cancer [13]
Oral (Drinking water)	Chronic (2-year study)	1000 ppm; 47 mg/kg bw/day	30	1.6 mg/kg-bw/day^c^	Bogdanffy et al. [17]

^a^AEGL-1 value.

^b^MRL.

^c^Toxicological threshold derived from POD reported in Bogdanffy et al. [17], using a UF of 30 [17] and assumption of 100% absorption [14].

Acute (i.e., daily) and chronic (i.e., annual+) inhalation exposure concentrations were compared to the general population ATSDR minimal risk levels (MRLs) for vinyl acetate, which were draft values at the time of this assessment [14]. The acute and intermediate inhalation MRLs were based a 28-day rat inhalation study [15] in which a variety of nasal cellular damage was observed, including necrosis and cellular proliferation, at 600 and 1000 ppm (no-observed adverse effect level (NOAEL) = 200 ppm, 704 mg/m^3^). The chronic MRL is based on a 2-year rat inhalation study [16] in which non-cancerous nasal lesions were observed at terminal sacrifice. An uncertainty factor of 30 was applied to account for species extrapolation [3] and human interspecies [11].

A chronic drinking water study by Bogdanffy et al. [17], which assessed chronic oral exposures to vinyl acetate in drinking water, was used in this study for comparison with absorbed doses integrated across multiple routes of exposure. Due to reduced water consumption and body weight gain at drinking water vinyl acetate concentrations of 5000 ppm, the NOAEL in the study was identified as the vinyl acetate exposure drinking water concentration of 1000 ppm; male rats in this exposure group had an estimated average daily intake of 47 mg/kg/day.

#### Exposure assessment modeling of products

In conducting the exposure modeling, standard assumptions for different exposure variables were used. These included an adult body weight of 68.8 kg and an adult inhalation rate of 25 L/min for the ConsExpo assessments. Furthermore, a 50% inhalation absorption rate and 100% dermal absorption rate were assumed for both ConsExpo and CEM assessments [1]. Health Canada noted that these conservative absorption rates result in a slightly higher modeled exposure. For all products where VAM was not detected, the concentration of VAM in the product was conservatively assumed to be equal to the LOD. The physical chemical properties of VAM that were used to model exposure are listed in Supplementary Table S1. Supplementary Tables S2–S12 contain all supporting information on the inputs used in the model scenarios described below.

##### Dietary supplement tablet

The first product evaluated is a dietary supplement in the form of coated tablets. Exposure estimates were made with the direct ingestion calculation below using a serving size of 4.2 g. This mass is based on analytical testing results that showed a single dietary supplement tablet averaged 1.40 g and the serving size listed on the packaging, which is three dietary supplement tablets per day. See Supplementary Table S2 for all model inputs and outputs.

$$\frac{{{{\bf{4.2}}}}\frac{{{{\boldsymbol{g}}}}}{{{{\boldsymbol{day}}}}}\,\times \,{{{\bf{0.0000001}}}}\,\times \,{{{\bf{1000}}}}\frac{{{{\boldsymbol{mg}}}}}{{{{\boldsymbol{g}}}}}}{{{{\bf{70}}}}{{{\boldsymbol{kg}}}}}$$

##### Lip gloss

The lip gloss product is a children’s product intended for use by children 6 years and older. Although there is a default scenario in ConsExpo for adult application of lipstick/lip salve, a more appropriate description of this product is in the Children’s Toys Fact Sheet for children applying lipstick as toys. This model reflects the use of the product “incidentally and not periodically” and includes the oral route of exposure only. All default inputs were assumed for this oral-only model as outlined in the Children’s Toys Fact Sheet with the exception of child body weight which was adjusted to reflect the 6–11-year age range [18]. Although inhalation and dermal were not included as potential routes of exposure, 100% of the VAM mass was assumed to be available for ingestion. See Supplementary Table S3 for all model inputs and outputs.

##### Face mask

The face mask product, which is intended to be left on the skin for a period and then washed off, was assessed with the face pack model in ConsExpo. All default inputs were assumed for this dermal-only model, as outlined in the cosmetics fact sheet [19]. Although inhalation and ingestion were not included as potential routes of exposure, 100% of the VAM mass was assumed to be available for dermal exposure. See Supplementary Table S4 for all model inputs and outputs.

##### Arts and crafts glue

The arts and crafts glue is a water-based multipurpose, all-in-one sealant, glue, and finish that was assessed with the bottled glue–universal/wood glue model in ConsExpo. Because the product can be used by both adults and children, two separate assessments were carried out.

All inputs remained the same between the adult and child assessments except for body weight (default 68.8 kg adult and 24.3 kg child 6–11 years old), inhalation rate (default 25 L/min adult and 0.762 m^3^/h child 6–11 years old)^1^, and exposed skin surface area (default 15 cm^2^ for two adult fingertips, 3.9 cm^2^ for two child fingertips). This assumes that the child uses the glue in the same manner as an adult.

A larger applied surface area and mass were substituted in place of the model defaults to account for the product’s use as a sealer and finish. These values came from the Do-It-Yourself products fact sheet, section 5.6 “Consumer behavior while using glue products (unpublished status 09/2022)” and Annex II, A.4, which describe the results of a consumer survey on exposure-related information during the use of different types of glues. The maximum product amount (25.0 g [Table 76]) and application duration (266 min [Table 77]) were assumed in the model and the release area was scaled up to account for the higher product amount (default is 10 g/0.05 m^2^ = 25 g/0.125 m^2^) [19]. See Supplementary Table S5 for all model inputs and outputs.

##### Joint compound

The joint compound product is a premixed paste product that is intended for interior ceiling and wall repair. The ConsExpo model, filler and putties—large hole filler—application was used to estimate potential exposure. All default inputs were assumed for this inhalation and dermal model except for the dilution assumption, as the product is premixed. It should also be noted that the mixing and loading step was also not included because the product is premixed. See Supplementary Table S6 for all model inputs and outputs.

##### Caulk

The caulk product is a latex-based caulking compound for both interior and exterior surfaces with a cure time of 72 h and was evaluated with the Joint Sealants model of ConsExpo. All default values were assumed as described in the Do-It-Yourself products fact sheet [20]. See Supplementary Table S7 for all model inputs and outputs.

##### Seam adhesive

The seam adhesive product is intended for sealing seams, edges, and tears in wallpaper and was assessed using the tube glue model in ConsExpo. All default values were assumed as described in the Do-It-Yourself products fact sheet [20]. See Supplementary Table S8 for all model inputs and outputs.

##### Concrete resurfacer

The concrete resurfacer product is a dry product that is intended to be used over the surface of existing concrete, on interior or exterior floors, and was assessed with the plasters and equalizers—floor equalizer— mixing and loading application in ConsExpo.

All default inputs were assumed for the mixing and loading scenario although according to the Do-It-Yourself products fact sheet, six intervals of mixing and loading per application are needed to mix the entire product amount because of the relatively fast hardening time. In addition, it was assumed that there are three applications of this product per year [20]. However, the model cannot be modified to account for multiple occurrences per day and per year. To address this, two separate mixing and loading scenarios were run: one that included six instances of mixing and loading in a single day, which was used to inform the acute/daily values and one that included 18 instances of mixing and loading in a year, which was used to inform the chronic/yearly values.

According to the Do-It-Yourself products fact sheet, “Inhalation exposure is not expected in this case because the floor equalizer does not contain ingredients that are likely to evaporate” [20]. However, our assumption is that the concrete resurfacer does contain VAM, which is considered volatile, so an evaporation-only inhalation model was added to supplement the default dermal application scenario. The additional inputs associated with this scenario are shown in the Inhalation Model section of Table S9 in the Supplementary Information. All default values for the dermal scenario were assumed as described in the Do-It-Yourself products fact sheet [20].

##### Primer

The primer product is a water-based wall and ceiling primer that was assessed with the water-based wall paint model of CEM. All default values were assumed for a “stay-at-home” adult using one gallon of paint. The following values were estimated using CEM’s internal calculations: saturation air concentration and skin permeability coefficient. Inputs to these estimates are included in Table S1 on VAM physical-chemical properties in the Supplementary Information. See Supplementary Table S10 for all model inputs and outputs.

##### Electronic tablet cover

The electronic tablet cover product is marketed as an electronic tablet casing intended for children and was assessed with the CEM model for plastic articles: other objects with potential for routine contact (toys, foam blocks, tents). It was assumed that the child directly mouths a 10 cm^2^ area of the electronic tablet cover, which is a “medium” surface area in CEM. The surface area of the product is based on the dimensions reported on the product website (~10 × 10 × 1 in.). A child’s dermal exposure duration (180 min) using the electronic tablet cover is based on the average time boys ages 3–11 spent watching television on the weekend (Table 9-1, child-specific exposure factors handbook [21]).

Except for the inputs listed next, all inputs were default values for a “small child” (age 3–5 years) who stays at home and uses the electronic tablet in a single session during the day. The following values were estimated using CEM’s internal calculations: initial concentration of semi-volatile organic compounds (SVOC) in the article, saturation air concentration, all dust parameter inputs, skin permeability coefficient, chemical migration rate (based on <0.1 ppm content), transdermal permeability coefficient, and average molecule diffusion per contact. Inputs to these estimates are included in Table S1 on VAM physical-chemical properties in the Supplementary Information. See Supplementary Table S11 for all model inputs and outputs.

##### Shelf liner

The shelf liner product is a non-adhesive cupboard shelf and drawer liner that comes in multiple sizes, the largest being 38.7 ft^2^, and was assessed using the plastic articles: vinyl flooring model in CEM. Except for the inputs listed next, all inputs were default values for an adult who stays at home and installs five shelf liners in a single day. The following values were estimated using CEM’s internal calculations: initial concentration of SVOC in the article, all dust parameter inputs, saturation concentration in air, skin permeability coefficient, chemical migration rate (based on <0.1 ppm content), transdermal permeability coefficient, and average molecule diffusion per contact. Inputs to these estimates are included in Table S1 on VAM physical-chemical properties in the Supplementary Information. See Supplementary Table S12 for all model inputs and outputs.

## Results

### Consumer product chemical analysis

Of the 71 consumer products analyzed, 40 had VAM concentrations less than the lowest detection limit achievable (0.1–2 ppmw) using the HS/GC method. Another 19 products had non-detectable VAM, but the product materials demonstrated atypical or nonlinear calibration behavior with the method. Four products had detectable VAM in the 2–10 ppmw range and eight had detectable VAM from 10 to 648 ppmw. A summary of the results from consumer product analysis is provided in Supplementary Table S13.

For many of the samples analyzed in Table S13, residual VAM was not detected, and the samples exhibited what would be considered typical calibration behavior and detection limits. That is, VAM was easily detected in samples spiked at the lowest calibration level (2 μg/μL, equivalent to 2 ppmw in a 1 g sample), and detection limits were approximately an order of magnitude lower than this standard.

VAM LOD was determined by analysis of a neat 2 μg/mL VAM liquid standard and the signal-to-noise ratio was determined using VAM peak area. The VAM peak area LOD was then determined by calculating the VAM peak area corresponding to a VAM peak with a S/N value of 3. This area was 0.0040 pA* min and was subsequently applied to all samples in which VAM was not detected. The VAM LOD for each individual sample was calculated using this area and the average response factor of the duplicate sample replicates spiked at the 2 μg/μL level.

Some samples in Table S13 exhibited atypical calibration behavior and detection limits (denoted with two asterisks). In one set of samples, VAM was not detected in either the sample or in the spiked calibration replicates at either the lowest (2 μg/μL, equivalent to 2 ppmw in a 1 g sample) or highest (200 μg/μL, equivalent to 200 ppmw in a 1 g sample) calibration levels. As a result, no LOD could be determined, which is indicated in the table as detection limit not available, or NA. In another set of samples, VAM was not detected in the sample, which is indicated by ND in the table, nor was VAM detected in the spiked calibration replicates at the lowest calibration level (2 ppmw). However, unlike the previous set, this set of samples did exhibit a detectable level of VAM in the spiked calibration replicates at the highest calibration level (200 ppmw). The LOD for these samples was therefore based upon the highest calibration level and the resulting LODs were generally unusually high, as might be expected. However, in a few cases, the LOD based upon the highest calibration level was sufficiently low (<1 ppmw) that VAM should have been detected at the lowest calibration level (2 ppmw). The fact that it was not detected at this level is an indication of nonlinear behavior. In fact, the last sample in the table is one in which VAM had been detected at >10 ppmw in the screening analysis, so a full calibration set was prepared and analyzed (see Supplementary Information).

For the samples marked with ‘^’ in Table S13, the unusual partitioning and/or calibration behavior is likely due to sorption or reaction of VAM within the sample matrix. Many of these samples were composed primarily of inorganic materials such as gypsum, and many of them contained water. Volatile organic compounds (VOCs) such as vinyl acetate are known to be absorbed or adsorbed onto porous inorganic substrates, and vinyl acetate is known to undergo acid-catalyzed hydrolysis. Regardless of the mechanism, such processes provide likely explanations for both the absence of any residual VAM in the samples themselves as well as the inability to detect any VAM in the equilibrium headspace of the spiked calibration replicates of these samples. While the inability to detect VAM in either of the spiked calibration replicates precludes the determination of a LOD for the residual VAM content of the corresponding samples, it does not preclude the determination of a LOD for the VAM from these samples themselves. The previously described analysis demonstrated that no detectable level of VAM is emitted from these samples under static conditions at 90 °C, and a LOD for VAM in the equilibrium headspace can be estimated to provide a LOD for the VAM emitted from these samples under these conditions. Using the data obtained from an analysis of the neat 2 μg/μL VAM liquid standard, it was estimated that VAM could be detected in the headspace at a concentration of ~0.003 μg/mL, equivalent to ~0.8 ppmw. Therefore, for each of the atypical samples described above in which a LOD for VAM content could not be determined, the value of 0.8 ppmw can be used as a LOD for VAM under static conditions at 90 °C.

### Consumer exposure and risk assessment

Seven products were evaluated with ConsExpo: lip gloss, face mask, arts and crafts glue, joint compound, caulk, seam adhesive, and concrete resurfacer; three products were evaluated with CEM: primer, electronic tablet cover, and shelf liner; and one product was evaluated with a direct oral intake calculation only: dietary supplement tablet.

The terminology used by ConsExpo and CEM to describe exposure estimate outputs are slightly different. With the exception of the dietary supplement tablet, lip gloss, face mask, and primer, the inhalation pathway was evaluated with three exposure time periods, peak (15 min), acute (24 h), and chronic (1 year). In ConsExpo, the peak air concentration (mg/m^3^) output was calculated for instantaneous release, constant release, and evaporation inhalation models using a time-weighted average of the highest 15 min of exposure over the exposure period. In CEM, the peak concentration (mg/m^3^) is the highest concentration experienced at any point during the day of use in the use environment. Mean daily air concentration values in ConsExpo are referred to as “mean concentration on day of exposure” (mg/m^3^), which is defined as the average air concentration over the day, accounting for the number of events on one day. CEM did not have an equivalent daily air output for the product category models evaluated, thus the primer category did not have a mean daily air concentration or acute inhalation MOS. Chronic air concentrations in ConsExpo are represented by the “inhalation year average concentration” values, which is the mean daily air concentration averaged over 1 year. Within CEM, this chronic air value is referred to as the “chronic steady-state air SVOC” for the solid article models (shelf liner and tablet cover) and “chronic average daily concentration zone 1” for the formulated product model (primer).

All product categories were assessed for chronic internal exposure, which is the sum of absorbed dose estimates from the oral, dermal, and inhalation routes. In ConsExpo, this was the “integrated internal year average dose” value, which is the daily absorbed dose (per kg body weight) from all routes of exposure averaged over 1 year. In CEM, this was the “chronic average daily dose” value, which is defined as the average daily absorbed dose from all routes of exposure calculated with age-group-specific exposure factors over the course of 1 year. For the dietary supplement tablet, the direct oral intake calculation is conservatively assumed to equate to an estimate of chronic oral exposure from daily use.

As shown in Table 4, all adult and child MOE values were greater than the target MOE of 100, and all adult and child MOS values were greater than the target MOS of 1. The 11 products evaluated are considered to have low VAM exposure during typical product use. Modeled products were selected from a group of other VAM-containing products based on their VAM content or other characteristics to be representative of an entire product use category. As such, these conclusions can be reasonably applied to other products with similar or lower VAM content in the same product use category. Supplementary Tables S2–S12 contain all supporting information on the outputs resulting from the model scenarios described above.

**Table 4:** Exposure estimates and margins of exposure (MOE) and margins of safety (MOS).

Product	Chronic internal dose (mg/kg bw/day)	MOE-Oral^e^	Peak air concentration	MOS-peak inhalation^f^	Mean daily air concentration	MOS-acute inhalation^g^	Chronic air concentration	MOS-chronic inhalation^h^
Target value		**≥1.0E** + **02**		**≥1.0E** + **00**		**≥1.0E** + **00**		**≥1.0E** + **00**
Dietary supplement tablet^a^	6.0E-06^b^	7.8E + 06	--	--	--	--	--	--
Lip gloss^a^	8.1E-09^c^	1.9E + 08	--	--	--	--	--	--
Face mask^a^	1.8E-05^c^	8.8E + 04	--	--	--	--	--	--
Arts and crafts glue –adult	8.9E-04^c^	1.8E + 03	2.7E-01 mg/m^3^ (7.7E-02 ppm)	8.9E + 01	3.1E-02 mg/m^3^ (8.8E-03 ppm)	1.1E + 02	3.1E-03 mg/m^3^ (8.8E-04 ppm)	3.4E + 02
Arts and crafts glue –child	1.4E-03^c^	1.1E + 03	2.7E-01 mg/m^3^ (7.7E-02 ppm)	8.9E + 01	3.1E-02 mg/m^3^ (8.8E-03 ppm)	1.1E + 02	3.1E-03 mg/m^3^ (8.8E-04 ppm)	3.4E + 02
Joint compound^a^	4.6E-04^c^	3.4E + 03	1.9E + 00 mg/m^3^ (5.4E-01 ppm)	1.3E + 01	2.1E-01 mg/m^3^ (6.0E-02 ppm)	1.7E + 01	1.7E-03 mg/m^3^ (4.8E-04 ppm)	6.2E + 02
Caulk^a^	7.9E-06^c^	2.0E + 05	1.5E-01 mg/m^3^ (4.3E-02 ppm)	1.6E + 02	3.0E-03 mg/m^3^ (8.5E-04 ppm)	1.2E + 03	2.5E-05 mg/m^3^ (7.1E-06 ppm)	4.2E + 04
Seam adhesive	6.2E-05^c^	2.5E + 04	2.7E-02 mg/m^3^ (7.7E-03 ppm)	8.8E + 02	2.1E-03 mg/m^3^ (6.0E-04 ppm)	1.7E + 03	2.0E-04 mg/m^3^ (5.7E-05 ppm)	5.2E + 03
Concrete resurfacer	8.3E-04^c^	1.9E + 03	1.8E + 01 mg/m^3^ (5.1E + 00 ppm)	1.3E + 00	3.5E-01 mg/m^3^ (9.9E-02 ppm)	1.0E + 01	2.9E-03 mg/m^3^ (8.2E-04 ppm)	3.6E + 02
Primer^a^	2.2E-05^d^	7.1E + 04	9.4E-04 mg/m^3^ (2.7E-04 ppm)	2.6E + 04	--	--	5.8E-06 mg/m^3^ (1.6E-06 ppm)	1.8E + 05
Tablet cover^a^	7.0E-03^d^	2.3E + 02	--	--	--	--	9.4E-03 mg/m^3^ (2.7E-03 ppm)	1.1E + 02
Shelf liner^a^	3.9E-03^d^	4.0E + 02	--	--	--	--	9.4E-03 mg/m^3^ (2.7E-03 ppm)	1.1E + 02

^a^Levels of VAM in this product were not detectable; modeling conservatively based on the limit of detection for each product.

^b^Modeling done by direct oral intake calculation: dose mg/kg bw/day.

^c^Modeling done in ConsExpo: integrated internal year average dose mg/kg/bw/day.

^d^Modeling done in CEM: chronic average daily dose mg/kg/day.

^e^Internal dose from all relevant routes of exposure compared to oral chronic (2-year study) POD of 47 mg/kg bw/day.

^f^Compared to acute 10-min POD of 24 mg/m^3^ (6.82 ppm).

^g^Compared to acute 1–14 days POD of 3.52 mg/m^3^ (1.00 ppm).

^h^Compared to chronic 1+ year POD of 1.06 mg/m^3^ (0.30 ppm).

--Route and time period of exposure not relevant based on model defaults.

Bolded values are the target values used in the MOE and MOS calculations.

## Discussion

Vinyl acetate polymers and copolymers are used in a variety of consumer products. Understanding the levels of residual VAM in these products and the potential exposure from their use is an important part of evaluating the risk of these consumer products. This paper presents an approach for measuring and evaluating consumer exposure to VAM from products in an array of product categories. A representative sample of products was chosen from each product category based on VAM content, product use characteristics, and target population. Because residual VAM was not detected in all products sampled, for the purpose of this exposure assessment, it was conservatively assumed that VAM was present in those products at the LOD. Consumer exposure was estimated with ConsExpo and CEM and compared to a series of acute and chronic regulatory guidance and toxicological thresholds.

An analytical method utilizing headspace (HS) vapor capture and GC was developed to analyze residual VAM in a variety of consumer products. The HS/GC is not applicable to materials containing significant quantities of components which are more volatile than water. Examples of such products include nail enamels and sealants in pressurized canisters. Several products exhibited unusual partitioning and/or calibration behavior likely due to sorption or reaction of VAM within the sample matrix, resulting in the inability to detect any VAM in the equilibrium headspace of the spiked calibration replicates of these samples. In such cases, a VAM detection limit was estimated based upon data obtained from an analysis of a neat VAM standard. The purpose of conducting the analysis using this particular methodology was to provide an accurate assessment of the VAM content in the original samples, independent of the type or form of sample. The accuracy of this method is not dependent upon the complete extraction of VAM from the sample. Additionally, excluding the possibility of decomposition of a particular sample, the use of elevated temperatures and extended equilibration times does not in itself lead to any systematic bias in the reported VAM content.

Products in the glue category had the highest levels of VAM, ranging from 1.0 to 648 ppmw. The two products with detectable levels of VAM in the surface treatment category were the next highest and ranged from 16 to 22 ppmw. Caulks/sealants had two products with detectable levels, ranging from 2.3 to 7.6 ppmw. Residual VAM was not detected in any of the products in the following categories: ingestibles, cosmetics/personal care, paint, and solid article. Due to the method limitations discussed above, there was wide variability in the LODs (<0.1 to <200 ppmw). There are few peer-reviewed studies in the published literature on VAM exposure. One occupational exposure study by Kominsky et al. [22] showed non-detectable levels of VAM in personal breathing zone and area samples collected during spray and roller application and drying of PVA emulsion paints in simulated closed and open room conditions. Detection limits for VAM ranged from 0.01 to 0.37 ppmw in the air and were significantly lower than the Occupational Safety and Health Administration’s 8-h permissible exposure limit of 10 ppm.

Interestingly, although the two solid article products (electronic tablet cover and shelf liner) had non-detectable levels of VAM, the MOE based on all routes of exposure was the lowest of all products (230 and 400, respectively, when the concentration in the product was assumed to be equal to the LOD limit). In the case of the electronic tablet cover, this is likely attributed to the assumptions in the model for small children (ages 3–5 years) who have the highest dust ingestion rate (100 mg/day) and the second highest mouthing duration (8.4 min/h) of all age groups. The electronic tablet cover is also assumed to have a relatively long direct dermal contact time of 3 h per day. Regarding the shelf liner, it was conservatively assumed that multiple units of the shelf liner product were installed in 1 day, thus leading to relatively elevated inhalation and dermal exposure estimates.

The product associated with the highest 15-min peak inhalation exposure and lowest MOS was the concrete resurfacer. In ConsExpo, the exposure was estimated with a spray model that assumes mixing and loading of the dry product takes place in six short increments of 3 min each, plus one 30-min application period, with the application period contributing most significantly to the high 15-min peak exposure. In reality, the full product amount (27 kg) would not be applied all at once and would instead be applied incrementally in conjunction with the series of six short mixing and loading periods. The 15-min peak inhalation air concentration during the application phase would be reduced by a factor of six, thus also increasing the MOS. All other MOS for acute and chronic inhalation endpoints were one or more orders of magnitude greater than the target MOS.

This exposure assessment was designed to approximate real-world VAM exposure to consumers from various products. Model scenarios were chosen that were representative of product use based on product use instructions. Uncertainty exists in how products will be used by consumers, and therefore, the variability in product use across all consumers is not captured in this deterministic assessment. Model default parameters were selected except where product- or consumer-specific parameters were necessary (e.g., concrete resurfacer evaporation exposure during application, child body weight for lip gloss product). In CEM, the “stay-at-home” scenario was conservatively chosen, which assumes the user will remain in the near-field^2^ or far-field^3^ exposure zones for a majority of the day.

Conservatism was also built in by assuming the full LOD as the VAM concentration for products with non-detectable levels of VAM. Furthermore, all VAM was assumed to be available for exposure, with no loss due to degradation or evaporation. However, just one product sample was used in the chemical analysis of VAM; thus, the variability of VAM across multiple samples of the same product was not captured in this assessment.

In conclusion, all representative product use scenarios were found to generate low exposure to VAM when compared to acute and chronic non-cancer regulatory and toxicological thresholds, with MOS and MOE exceeding the target of 1 and 100, respectively. Products with large surface area (shelf liner), high contact time (electronic tablet cover), and high product amount (concrete resurfacer) led to the highest potential VAM exposures. These exposures were primarily driven by the inhalation route due to VAM’s high vapor pressure and volatile nature. This is the only study to date to report measured levels of VAM in consumer products and the implications of those levels on consumer exposure. Actual exposure to VAM during product use will vary depending on how the product is used and under what conditions. There is also a potential for additional consumer exposure to VAM through other products or sources not covered in this paper, such as food that contains VAM as a starch modifier or food packaging products [14].

Further verification and refinement of the analytical method would help address some of the limitations in measuring VAM in certain matrices. Should additional products in new product categories be identified, modeling should be repeated to evaluate the potential for consumer exposure as it relates to the acute and chronic regulatory and toxicological thresholds identified herein.

## Supplementary information

Supplementary figure

Supplementary tables

## Data Availability

All data needed to re-create these analyses are included in the Supplementary Information Tables S2–S13. Additional analytical instrument conditions and results are included in the Analytical Chemistry Supplementary Information.

## Footnotes

1 Default inhalation rates for both the adult and child scenario were calculated using body weight and “light exercise” within ConsExpo.

2 Defined as Zone 1 representing the room where the consumer product is used.

3 Defined as Zone 2 representing the remainder of the building of use.
