# Supplementary material for: Measurement of vinyl acetate monomer in consumer products and modeled estimates of consumer exposure
Source: J Expo Sci Environ Epidemiol. 2025 Jun 18;35(6):933–42. doi: 10.1038/s41370-025-00786-y (PMC12583140; doi:10.1038/s41370-025-00786-y)
Supplement: Supplementary file 1 — Supplementary figure [file 41370_2025_786_MOESM1_ESM.docx]

**Measurement of Vinyl Acetate Monomer in Consumer Products and Modeled Estimates of Consumer Exposure**

**Supplementary Information: Analytical Method Supporting Data**

Section 1. Chromatogram Showing Heartcut


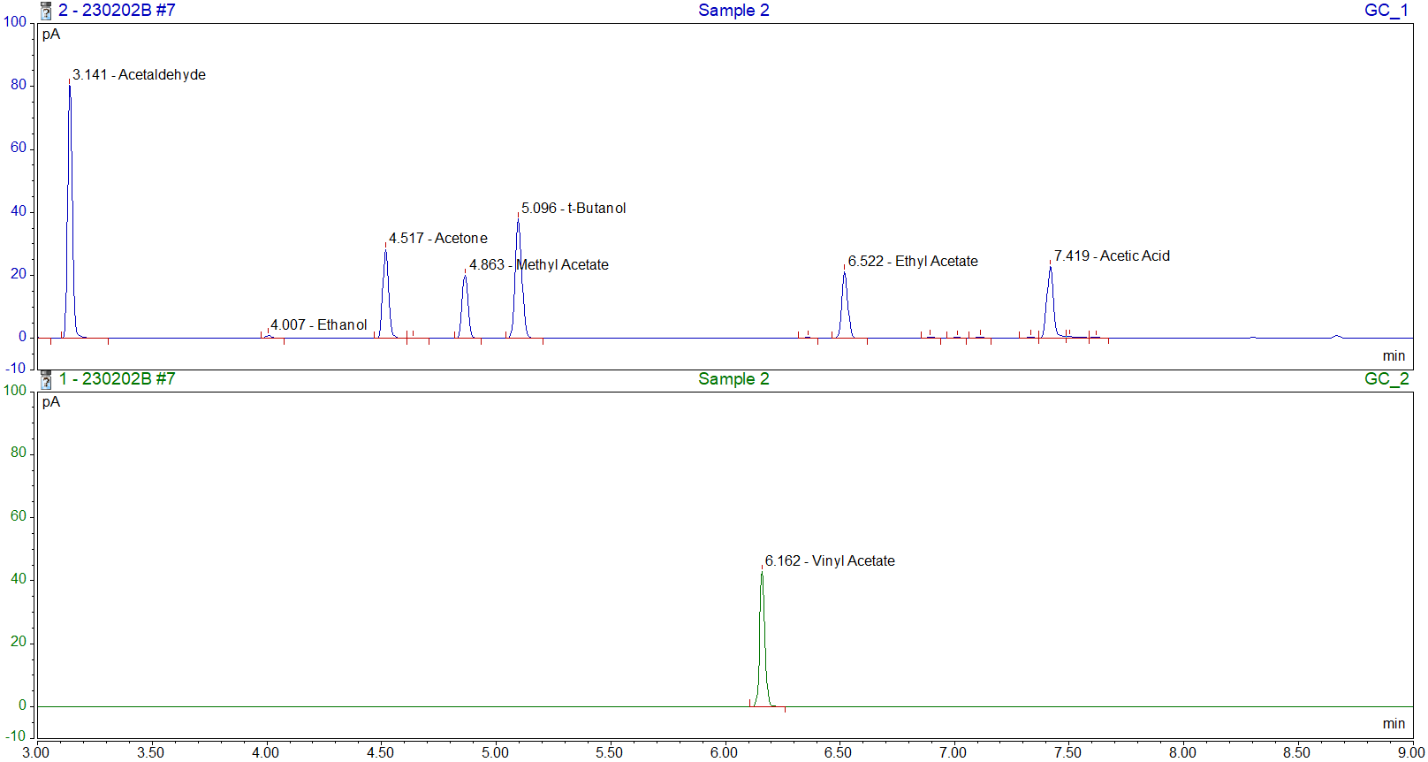


Figure S1 - Chromatograms from the analysis of a sample containing 50ppmw residual VAM, heartcut collection at 5.78-5.98mins, elution of VAM from the secondary column (Stabilwax) with detection via the secondary FID (GC_2, bottom chromatogram)

Section 2. Example Calibration Plots


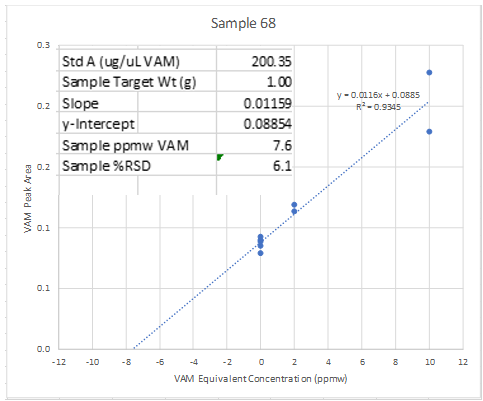


Figure S2 - Results from the analysis of sample 68, VAM detected at 2-10ppmw


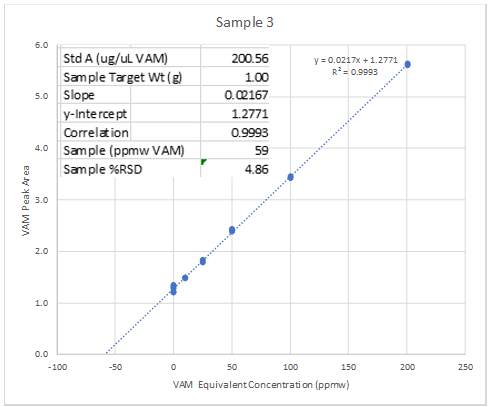


Figure S3 - Results from the analysis of sample 3, VAM detected at >10ppmw

Section 3. Instrument Conditions

- HS Instrument: Agilent Model G1888 Static Headspace Sampler
- Carrier Gas: Helium (supplied from GC EPC)
- Loop Volume: 1.0 mL
- Vial Volume: 20 mL (nominal)
- Vial Oven Temp: 90°C
- Valve/Loop Temp: 100°C (10°C above vial oven temperature)
- Transfer Line Temp: 150°C
- Vial Equil Time: 240 mins
- GC Cycle Time: 30 mins
- Injection Mode: Single
- Pressurization Gas: Nitrogen
- Vial Fill Pressure: 20.0 psig
- Vial Fill Time: 0.20 mins
- Vial Vent Time: 0.20 mins
- Loop Equil Time: 0.05 mins
- Inject Time: 0.50 mins
- GC Instrument: Agilent Model 7890 Gas Chromatograph
- Carrier Gas: Helium
- Inlet Type: Split/Splitless (SSL) Inlet
- Inlet Mode: Split mode, programmed pressure
- Inlet Temperature: 150°C
- Inlet Split Flow: 75 mL/min (split ratio 50:1 relative to primary column flow)
- Primary Column: 30 m x 0.25 mm ID 1.4 μm Restek Rtx-624 (bonded 6% cyanopropyl/phenyl, 94% polydimethylsiloxane) fused silica capillary column
- Primary Col Flow: 1.50 mL/min constant flow via programmed pressure
- Primary Restrictor: 26.0 cm x 0.10 mm ID fused silica capillary tubing
- Primary Restrictor Flow: 2.50 mL/min constant flow via programmed pressure
- Primary Pressure: 22.29 psig isobaric for 2.0 mins
- +0.8450 psig/min to 27.36 psig with no hold time
- +1.6314 psig/min to 38.78 psig and hold for 5.0 mins
- Secondary Column: 10 m x 0.25 mm ID 0.5μm Restek Stabilwax (bonded polyethylene glycol) fused silica capillary column
- Secondary Col Flow: 2.50 mL/min constant flow via programmed pressure
- Secondary Pressure: 10.64 psig isobaric for 2.0 mins
- +0.4383 psig/min to 13.27 psig with no hold time
- +0.8800 psig/min to 19.43 psig and hold for 5.0 mins
- Column Temperature: 40°C isothermal for 2.0 mins
- +10°C/min to 100°C with no hold time
- +20°C/min to 240°C and hold for 5.0 mins
- Primary Detector: Flame ionization detector
- Primary Det Temp: 260°C
- Primary Det Flows: 30 mL/min Hydrogen, 300 mL/min Air, 27.5 mL/min Nitrogen make-up gas
- Secondary Detector: Flame ionization detector
- Secondary Det Temp: 260°C
- Secondary Det Flows: 30 mL/min Hydrogen, 300 mL/min Air, 27.5 mL/min Nitrogen make-up gas
- Heartcut Time: 5.780–5.980 mins
- Data System: ThermoScientific Chromeleon v7.2.10 was used for instrument control, data acquisition and data processing
- Flow Control: PressProg v1.31, a custom-written Java desktop application, was used to determine the pressure program required to maintain constant flow conditions during column temperature programming using the Dean’s Switch

Section 4. Equilibration Studies


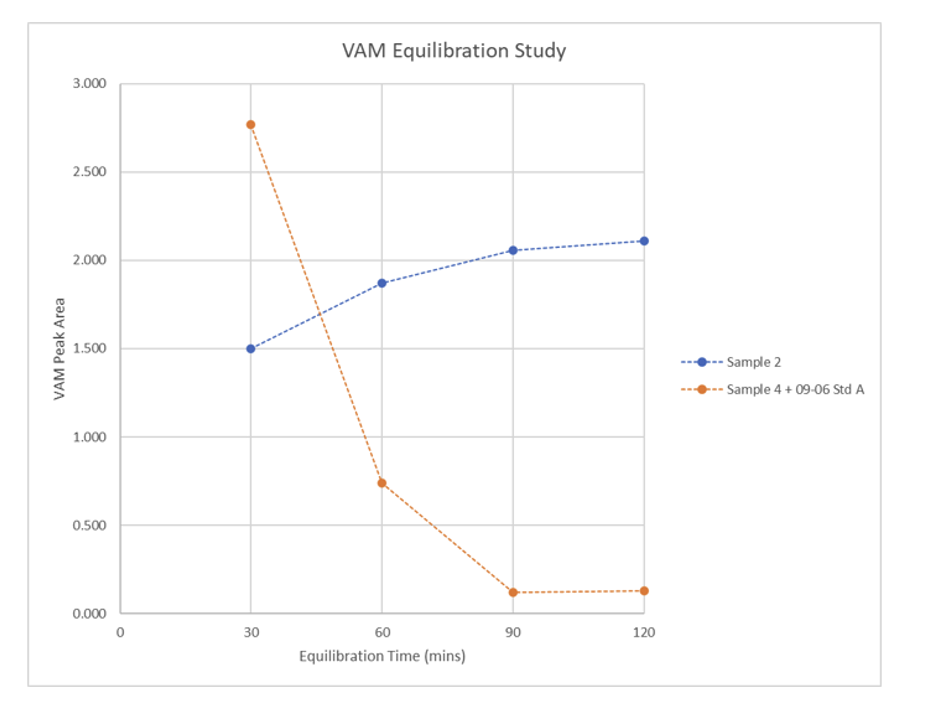


Figure S4. Representative equilibration kinetics for samples of different physical properties. Sample 2 was a glue product and Sample 4 was a spackle product
