# Supplementary material for: Measurement of vinyl acetate monomer in consumer products and modeled estimates of consumer exposure
Source: J Expo Sci Environ Epidemiol. 2025 Jun 18;35(6):933–42. doi: 10.1038/s41370-025-00786-y (PMC12583140; doi:10.1038/s41370-025-00786-y)
Supplement: Supplementary file 2 — Supplementary tables [file 41370_2025_786_MOESM2_ESM.docx]

**Measurement of Vinyl Acetate Monomer in Consumer Products and Modeled Estimates of Consumer Exposure**

**Supplementary Information: Input and Output Tables for all Product Models**

**S1 – Physical Chemical Properties of Vinyl Acetate Monomer**

**S2 – Input and Outputs for Tablet Direct Oral Intake Calculation**

**S3 – Input and Outputs for Lip Gloss in ConsExpo**

**S4 – Input and Outputs for Face Mask in ConsExpo**

**S5 – Input and Outputs for Arts & Crafts Glue in ConsExpo**

**S6 – Input and Outputs for Joint Compound in ConsExpo**

**S7 – Input and Outputs for Caulk in ConsExpo**

**S8 – Input and Outputs for Seam Adhesive in ConsExpo**

**S9 – Input and Outputs for Concrete Resurfacer in ConsExpo**

**S10 – Input and Outputs for Primer in CEM**

**S11 – Input and Outputs for Tablet Cover in CEM**

**S12 – Input and Outputs for Shelf Liner in CEM**

**S13 – Summary of VAM concentrations in consumer products, with estimated detection limits**

**References**

**Supplemental Table S1 – Physical Chemical Properties of Vinyl Acetate Monomer**

| **Property** | **Units** | **Value** | **Reference** |
| --- | --- | --- | --- |
| **Molecular Weight** | g/mol | 86.1 |  |
| **Melting Point** | ^°^C | -93.2 | 1 |
| **Boiling Point** | ^°^C | 72.7 | 2,3 |
| **Density** | g/cm^3^ | 0.932 | 2 |
| **Water Solubility** | mg/L | 2.00E+04 | 4 |
| **Vapour Pressure** | mm Hg | 115.5 | 5 (p. 2478) |
| **VP (Pa)** | pa | 15398.736 | Converted |
| **VP (torr)** | torr | 115.5 | Converted |
| **Henry’s Law constant, K_H_** | atm·m^3^/mol | 4.81E-04 | 6 |
| **Log K_H_** |  | -3.31 | Calculated from K_H_ |
| **Log Kow** |  | 0.73 | 5 (p. 1075) |
| **Log Koc** |  | 0.788-1.78 | 7,8 |
| **Log Koa** |  | 2.44 | Modeled with EIPSUITE KOAWIN (modeled 11/3/2023) |

**Supplemental Table S2 – Input and Outputs for Tablet Direct Oral Intake Calculation**

| **Input** | **Units** | **Tablet** |
| --- | --- | --- |
| **Product Mass** | g/day | 4.2 |
| **Concentration** | ppmw | 0.1^a^ |
| **Conversion** | mg/g | 1,000 |
| **Body weight** | kg | 70 |
| **Oral intake** | mg/kg bw/day | 6.00E-06 |
| ^a^Analytical result was non-detect (<0.1 ppmw) | | |

**Supplemental Table S3 – Input and Outputs for Lip Gloss in ConsExpo**

| **Input** | **Units** | **Lip Gloss - Child** |
| --- | --- | --- |
| **General Exposure Factors** | | |
| **Weight fraction substance** | % | 2.00E-05 |
| **Name** |  | ConsExpo fact sheet child 6-11 years |
| **Body weight** | kg | 24.3 |
| **Frequency** | per year | 1.20E+01 |
| **Oral Model** | | |
| **Exposure model** |  | Direct product contact – Direct oral intake |
| **Weight fraction substance** | % | 2.00E-5 |
| **Amount ingested** | g | 3.00E-2 |
| **Absorption model** |  | Fixed fraction |
| **Absorption fraction** |  | 1 |
| **Results: Oral** | | |
| **External event dose** | mg/kg bw | 2.47E-07 |
| **External dose on day of exposure** | mg/kg bw | 2.47E-07 |
| **Internal event dose** | mg/kg bw | 2.47E-07 |
| **Internal dose on day of exposure** | mg/kg bw/day | 2.47E-07 |
| **Internal year average dose** | mg/kg bw/day | 8.12E-09 |
| **Results: Integrated** | | |
| **Internal event dose** | mg/kg bw | 2.47E-07 |
| **Internal dose on day of exposure** | mg/kg bw/day | 2.47E-07 |
| **Internal year average dose** | mg/kg bw/day | 8.12E-09 |

**Supplemental Table S4 – Input and Outputs for Face Mask in ConsExpo**

| **Input** | **Units** | **Face Mask - Adult** |
| --- | --- | --- |
| **General Exposure Factors** | | |
| **Weight fraction substance** | % | 2.00E-05 |
| **Name** |  | ConsExpo fact sheet woman |
| **Body weight** | kg | 64.1 |
| **Frequency** | per year | 104 |
| **Dermal Model** | | |
| **Exposure model** |  | Direct contact – Instant application |
| **Exposed area** | cm^2^ | 565 |
| **Weight fraction substance** | % | 0.00002 |
| **Product amount** | g | 20 |
| **Contact Rate** | mg/min | -- |
| **Release duration** | min | -- |
| **Retention factor** |  | 1 |
| **Absorption model** |  | Fixed fraction |
| **Absorption fraction** |  | 1 |
| **Results: Dermal** | | |
| **Dermal load** | mg/cm^2^ | 7.08E-06 |
| **External event dose** | mg/kg bw | 6.24E-05 |
| **External dose on day of exposure** | mg/kg bw | 6.24E-05 |
| **Internal event dose** | mg/kg bw | 6.24E-05 |
| **Internal dose on day of exposure** | mg/kg bw/day | 6.24E-05 |
| **Internal year average dose** | mg/kg bw/day | 1.78E-05 |
|  |  |  |
| **Results: Integrated** | | |
| **Internal event dose** | mg/kg bw | 6.24E-05 |
| **Internal dose on day of exposure** | mg/kg bw/day | 6.24E-05 |
| **Internal year average dose** | mg/kg bw/day | 1.78E-05 |

**Supplemental Table S5 – Input and Outputs for Arts & Crafts Glue in ConsExpo**

| **Input** | **Units** | **Arts & Crafts Glue - Adult** | **Arts & Crafts Glue - Child** |
| --- | --- | --- | --- |
| **General Exposure Factors** | | | |
| **Weight fraction substance** | % | 0.0648 | 0.0648 |
| **Fact sheet** |  | ConsExpo fact sheet adult | ConsExpo fact sheet child 6-11 years |
| **Body weight** | kg | 68.8 | 24.3 |
| **Frequency** | per year | 36 | 36 |
| **Inhalation Model** | | | |
| **Exposure model** | Exposure to vapor-Evaporation | | |
| **Exposure duration** | minute | 240 | 240 |
| **Product in pure form** |  | No | No |
| **Molecular weight matrix** | g/mol | 3.00E+03 | 3.00E+03 |
| **The product is used in dilution** |  | No | No |
| **Product amount** | g | 25 | 25 |
| **Weight fraction substance** | % | 0.0648 | 0.0648 |
| **Room volume** | m^3^ | 20 | 20 |
| **Ventilation rate** | per hour | 0.6 | 0.6 |
| **Inhalation rate** |  | 25 l/min | 0.762 m3/hr |
| **Application temperature** | C | 20 | 20 |
| **Vapor pressure** | Pa | 1.54E+04 | 1.54E+04 |
| **Molecular weight** | g/mol | 86.1 | 86.1 |
| **Mass transfer coefficient** | m/hr | 10 | 10 |
| **Release area mode** |  | Increasing | Increasing |
| **Release area** | m^2^ | 0.125 | 0.125 |
| **Application duration** | minute | 266 | 266 |
| **Absorption model** |  | Fixed fraction | Fixed fraction |
| **Absorption fraction** |  | 0.5 | 0.5 |
| **Dermal Model** | | | |
| **Exposure model** |  | Direct contact - Instant application | |
| **Exposed area** | cm^2^ | 1.50E+01 | 3.90E+00 |
| **Weight fraction substance** | % | 0.0648 | 0.0648 |
| **Product amount** | g | 0.08 | 0.08 |
| **Retention factor** |  | 1 | 1 |
| **Absorption model** |  | Fixed fraction | Fixed fraction |
| **Absorption fraction** |  | 1 | 1 |
| **Results: Inhalation** | | | |
| **Mean event concentration** | mg/m^3^ | 1.89E-01 | 1.89E-01 |
| **Peak concentration (TWA 15 min)** | mg/m^3^ | 2.71E-01 | 2.71E-01 |
| **Mean concentration on day of exposure** | mg/m^3^ | 3.14E-02 | 3.14E-02 |
| **Year average concentration** | mg/m^3^ | 3.10E-03 | 3.10E-03 |
| **External event dose** | mg/kg bw | 1.64E-02 | 2.37E-02 |
| **External dose on day of exposure** | mg/kg bw | 1.64E-02 | 2.37E-02 |
| **Internal event dose** | mg/kg bw | 8.22E-03 | 1.18E-02 |
| **Internal dose on day of exposure** | mg/kg bw/day | 8.22E-03 | 1.18E-02 |
| **Internal year average dose** | mg/kg bw/day | 8.11E-04 | 1.17E-03 |
| **Results: Dermal** | | | |
| **Dermal load** | mg/cm^2^ | 3.46E-03 | 1.33E-02 |
| **External event dose** | mg/kg bw | 7.53E-04 | 2.13E-03 |
| **External dose on day of exposure** | mg/kg bw | 7.53E-04 | 2.13E-03 |
| **Internal event dose** | mg/kg bw | 7.53E-04 | 2.13E-03 |
| **Internal dose on day of exposure** | mg/kg bw/day | 7.53E-04 | 2.13E-03 |
| **Internal year average dose** | mg/kg bw/day | 7.43E-05 | 2.10E-04 |
| **Results: Integrated** | | | |
| **Internal event dose** | mg/kg bw | 8.98E-03 | 1.40E-02 |
| **Internal dose on day of exposure** | mg/kg bw/day | 8.98E-03 | 1.40E-02 |
| **Internal year average dose** | mg/kg bw/day | 8.85E-04 | 1.38E-03 |

**Supplemental Table S6 – Input and Outputs for Joint Compound in ConsExpo**

| **Input** | **Units** | **Joint Compound - Adult** |
| --- | --- | --- |
| **General Exposure Factors** | | |
| **Weight fraction substance** | % | 0.02 |
| **Fact sheet** |  | ConsExpo fact sheet adult |
| **Body weight** | kg | 68.8 |
| **Frequency** | per year | 3 |
| **Inhalation Model** | | |
| **Exposure model** |  | Exposure to vapor- evaporation |
| **Exposure duration** | minute | 240 |
| **Product in pure form** |  | No |
| **Molecular weight matrix** | g/mol | 3.00E+03 |
| **The product is used in dilution** |  | No |
| **Product amount** | g | 375 |
| **Weight fraction substance** | % | 0.02 |
| **Room volume** | m^3^ | 20 |
| **Ventilation rate** | per hour | 0.6 |
| **Inhalation rate** |  | 25 l/min |
| **Application temperature** | C | 20 |
| **Vapor pressure** | Pa | 1.54E+04 |
| **Molecular weight** | g/mol | 86.1 |
| **Mass transfer coefficient** | m/hr | 10 |
| **Release area mode** |  | Increasing |
| **Release area** | m^2^ | 0.0025 |
| **Application duration** | minute | 30 |
| **Absorption model** |  | Fixed fraction |
| **Absorption fraction** |  | 0.5 |
| **Dermal Model** | | |
| **Exposure model** |  | Direct contact - Instant application |
| **Exposed area** | cm^2^ | 4.50E+02 |
| **Weight fraction substance** | % | 0.02 |
| **Product amount** | g | 0.4 |
| **Retention factor** |  | 1 |
| **Absorption model** |  | Fixed fraction |
| **Absorption fraction** |  | 1 |
| **Results: Inhalation** | | |
| **Mean event concentration** | mg/m^3^ | 1.25E+00 |
| **Peak concentration (TWA 15 min)** | mg/m^3^ | 1.85E+00 |
| **Mean concentration on day of exposure** | mg/m^3^ | 2.08E-01 |
| **Year average concentration** | mg/m^3^ | 1.71E-03 |
| **External event dose** | mg/kg bw | 1.09E-01 |
| **External dose on day of exposure** | mg/kg bw | 1.09E-01 |
| **Internal event dose** | mg/kg bw | 5.45E-02 |
| **Internal dose on day of exposure** | mg/kg bw/day | 5.45E-02 |
| **Internal year average dose** | mg/kg bw/day | 4.48E-04 |
| **Results: Dermal** | | |
| **Dermal load** | mg/cm^2^ | 1.78E-04 |
| **External event dose** | mg/kg bw | 1.16E-03 |
| **External dose on day of exposure** | mg/kg bw | 1.16E-03 |
| **Internal event dose** | mg/kg bw | 1.16E-03 |
| **Internal dose on day of exposure** | mg/kg bw/day | 1.16E-03 |
| **Internal year average dose** | mg/kg bw/day | 9.56E-06 |
| **Results: Integrated** | | |
| **Internal event dose** | mg/kg bw | 5.57E-02 |
| **Internal dose on day of exposure** | mg/kg bw/day | 5.57E-02 |
| **Internal year average dose** | mg/kg bw/day | 4.58E-04 |

**Supplemental Table S7 – Input and Outputs for Caulk in ConsExpo**

| **Input** | **Units** | **Caulk - Adult** |
| --- | --- | --- |
| **General Exposure Factors** | | |
| **Weight fraction substance** | % | 0.00076 |
| **Name** |  | ConsExpo fact sheet adult |
| **Body weight** | kg | 68.8 |
| **Frequency** | per year | 3 |
| **Inhalation Model** | | |
| **Exposure model** |  | Exposure to vapour-Evaporation |
| **Exposure duration** | minute | 45 |
| **Product in pure form** |  | No |
| **Molecular weight matrix** | g/mol | 3.00E+03 |
| **The product is used in dilution** |  | No |
| **Product amount** | g | 350 |
| **Weight fraction substance** | % | 0.00076 |
| **Room volume** | m^3^ | 10 |
| **Ventilation rate** | per hour | 2 |
| **Inhalation rate** | l/min | 25 |
| **Application temperature** | C | 20 |
| **Vapor pressure** | Pa | 1.54E+04 |
| **Molecular weight** | g/mol | 86.1 |
| **Mass transfer coefficient** | m/hr | 10 |
| **Release area mode** |  | Increasing |
| **Release area** | m^2^ | 0.025 |
| **Application duration** | minute | 30 |
| **Absorption model** |  | Fixed fraction |
| **Absorption fraction** |  | 0.5 |
| **Dermal Model** | | |
| **Exposure model** |  | Direct contact- Constant Rate |
| **Exposed area** | cm^2^ | 3.00E+01 |
| **Weight fraction substance** | % | 0.00076 |
| **Product amount** | g | -- |
| **Contact Rate** | mg/min | 50 |
| **Release duration** | min | 30 |
| **Retention factor** |  | 1 |
| **Absorption model** |  | Fixed fraction |
| **Absorption fraction** |  | 1 |
| **Results: Inhalation** | | |
| **Mean event concentration** | mg/m^3^ | 9.74E-02 |
| **Peak concentration (TWA 15 min)** | mg/m^3^ | 1.47E-01 |
| **Mean concentration on day of exposure** | mg/m^3^ | 3.04E-03 |
| **Year average concentration** | mg/m^3^ | 2.50E-05 |
| **External event dose** | mg/kg bw | 1.59E-03 |
| **External dose on day of exposure** | mg/kg bw | 1.59E-03 |
| **Internal event dose** | mg/kg bw | 7.96E-04 |
| **Internal dose on day of exposure** | mg/kg bw/day | 7.96E-04 |
| **Internal year average dose** | mg/kg bw/day | 6.54E-06 |
| **Results: Dermal** | | |
| **Dermal load** | mg/cm^2^ | 3.80E-04 |
| **External event dose** | mg/kg bw | 1.66E-04 |
| **External dose on day of exposure** | mg/kg bw | 1.66E-04 |
| **Internal event dose** | mg/kg bw | 1.66E-04 |
| **Internal dose on day of exposure** | mg/kg bw/day | 1.66E-04 |
| **Internal year average dose** | mg/kg bw/day | 1.36E-06 |
| **Results: Integrated** | | |
| **Internal event dose** | mg/kg bw | 9.62E-04 |
| **Internal dose on day of exposure** | mg/kg bw/day | 9.62E-04 |
| **Internal year average dose** | mg/kg bw/day | 7.91E-06 |

**Supplemental Table S8 – Input and Outputs for Seam Adhesive in ConsExpo**

| **Input** | **Units** | **Seam Adhesive - Adult** |
| --- | --- | --- |
| **General Exposure Factors** | | |
| **Weight fraction substance** | % | 0.0074 |
| **Fact sheet** |  | ConsExpo fact sheet adult |
| **Body weight** | kg | 68.8 |
| **Frequency** | per year | 36 |
| **Inhalation Model** | | |
| **Exposure model** |  | Exposure to vapor-Evaporation |
| **Exposure duration** | minute | 240 |
| **Product in pure form** |  | No |
| **Molecular weight matrix** | g/mol | 3.00E+03 |
| **The product is used in dilution** |  | No |
| **Product amount** | g | 9 |
| **Weight fraction substance** | % | 0.0074 |
| **Room volume** | m^3^ | 20 |
| **Ventilation rate** | per hour | 0.6 |
| **Inhalation rate** |  | 25 l/min |
| **Application temperature** | C | 20 |
| **Vapor pressure** | Pa | 1.54E+04 |
| **Molecular weight** | g/mol | 86.1 |
| **Mass transfer coefficient** | m/hr | 10 |
| **Release area mode** |  | Increasing |
| **Release area** | m^2^ | 0.02 |
| **Application duration** | minute | 36 |
| **Absorption model** |  | Fixed fraction |
| **Absorption fraction** |  | 0.5 |
| **Dermal Model** | | |
| **Exposure model** |  | Direct contact - Instant application |
| **Exposed area** | cm^2^ | 1.50E+01 |
| **Weight fraction substance** | % | 0.0074 |
| **Product amount** | g | 0.08 |
| **Retention factor** |  | 1 |
| **Absorption model** |  | Fixed fraction |
| **Absorption fraction** |  | 1 |
| **Results: Inhalation** | | |
| **Mean event concentration** | mg/m^3^ | 1.23E-02 |
| **Peak concentration (TWA 15 min)** | mg/m^3^ | 2.72E-02 |
| **Mean concentration on day of exposure** | mg/m^3^ | 2.06E-03 |
| **Year average concentration** | mg/m^3^ | 2.03E-04 |
| **External event dose** | mg/kg bw | 1.08E-03 |
| **External dose on day of exposure** | mg/kg bw | 1.08E-03 |
| **Internal event dose** | mg/kg bw | 5.38E-04 |
| **Internal dose on day of exposure** | mg/kg bw/day | 5.38E-04 |
| **Internal year average dose** | mg/kg bw/day | 5.31E-05 |
| **Results: Dermal** | | |
| **Dermal load** | mg/cm^2^ | 3.95E-04 |
| **External event dose** | mg/kg bw | 8.60E-05 |
| **External dose on day of exposure** | mg/kg bw | 8.60E-05 |
| **Internal event dose** | mg/kg bw | 8.60E-05 |
| **Internal dose on day of exposure** | mg/kg bw/day | 8.60E-05 |
| **Internal year average dose** | mg/kg bw/day | 8.49E-06 |
| **Results: Integrated** | | |
| **Internal event dose** | mg/kg bw | 6.25E-04 |
| **Internal dose on day of exposure** | mg/kg bw/day | 6.25E-04 |
| **Internal year average dose** | mg/kg bw/day | 6.16E-05 |

**Supplemental Table S9 – Input and Outputs for Concrete Resurfacer in ConsExpo**

| **Input** | **Units** | **Mixing and Loading 18x year –**  **Adult** | **Mixing and Loading 6x day - Adult** | **Application - Adult** |
| --- | --- | --- | --- | --- |
| **General Exposure Factors** | | | | |
| **Weight fraction substance** | % | 0.0016 | 0.0016 | 0.0016 |
| **Name** |  | ConsExpo Fact Sheet Adult | ConsExpo Fact Sheet Adult | ConsExpo Fact Sheet Adult |
| **Body weight** | kg | 68.8 | 68.8 | 68.8 |
| **Scenario** |  | Mixing and Loading 18x year | Mixing and Loading 6x day | Application |
| **Frequency** | per year (18, 3) per day (6) | 18 | 6 | 3 |
| **Inhalation Model** | | | | |
| **Exposure model** |  | Exposure to spray - Instantaneous release | Exposure to spray - Instantaneous release | Exposure to vapour - Evaporation |
| **Exposure duration** | minute | 3 | 3 | 30 |
| **Product in pure form** |  | No | No | No |
| **Molecular weight matrix** |  |  |  | 3.00E+03 |
| **The product is used in dilution** |  | No | No | No |
| **Released mass** | g | 0.000208 | 0.000208 |  |
| **Product amount** | g |  |  | 2.70E+04 |
| **Weight fraction substance** | % | 0.0016 | 0.0016 | 0.0016 |
| **Room volume** | m^3^ | 1 | 1 | 20 |
| **Ventilation Rate** | per hour | 0.6 | 0.6 | 0.6 |
| **Inhalation rate** | l/min | 25 | 25 | 25 |
| **Application temperature** | deg C |  |  | 20 |
| **Vapour pressure** | Pa |  |  | 1.54E+04 |
| **Molecular weight** | g/mol |  |  | 86.1 |
| **Mass transfer coefficient** | m/hr |  |  | 10 |
| **Release area mode** |  |  |  | Constant |
| **Release area** | m^2^ |  |  | 8 |
| **Emission duration** | minute |  |  | 30 |
| **Absorption model** |  | Fixed fraction | Fixed fraction | Fixed fraction |
| **Absorption fraction** | Fraction | 0.5 | 0.5 | 0.5 |
| **Dermal Model** | | | | |
| **Exposure model** |  | Direct contact - Constant rate | Direct contact - Constant rate | Direct contact - Instant application |
| **Exposed area** | cm^2^ | 4.50E+02 | 4.50E+02 | 9.00E+02 |
| **Weight fraction substance** | % | 0.0016 | 0.0016 | 0.0016 |
| **Contact rate** | mg/min | 5 | 5 |  |
| **Release duration** | minute | 3 | 3 |  |
| **Product amount** | g |  |  | 35 |
| **Retention factor** |  | 1 | 1 | 1 |
| **Absorption model** |  | Fixed fraction | Fixed fraction | Fixed fraction |
| **Absorption fraction** | Fraction | 1 | 1 | 1 |
| **Results: Inhalation** | | | | |
| **Mean event concentration** | mg/m^3^ | 3.28E-06 | 3.28E-06 | 1.70E+01 |
| **Peak concentration (TWA 15 min)** | mg/m^3^ | 3.28E-06 | 3.28E-06 | 1.83E+01 |
| **Mean concentration on day of exposure** | mg/m^3^ | 6.83E-09 | 4.10E-08 | 3.53E-01 |
| **Year average concentration** | mg/m^3^ | 3.37E-10 | 4.10E-08 | 2.91E-03 |
| **External event dose** | mg/kg bw | 3.57E-09 | 3.57E-09 | 1.85E-01 |
| **External dose on day of exposure** | mg/kg bw | 3.57E-09 | 2.14E-08 | 1.85E-01 |
| **Internal event dose** | mg/kg bw | 1.79E-09 | 1.79E-09 | 9.25E-02 |
| **Internal dose on day of exposure** | mg/kg bw/day | 1.79E-09 | 1.07E-08 | 9.25E-02 |
| **Internal year average dose** | mg/kg bw/day | 8.81E-11 | 1.07E-08 | 7.60E-04 |
| **Results: Dermal** | | | | |
| **Dermal load** | mg/cm^2^ | 5.33E-07 | 5.33E-07 | 6.22E-04 |
| **External event dose** | mg/kg bw | 3.49E-06 | 3.49E-06 | 8.14E-03 |
| **External dose on day of exposure** | mg/kg bw | 3.49E-06 | 2.09E-05 | 8.14E-03 |
| **Internal event dose** | mg/kg bw | 3.49E-06 | 3.49E-06 | 8.14E-03 |
| **Internal dose on day of exposure** | mg/kg bw/day | 3.49E-06 | 2.09E-05 | 8.14E-03 |
| **Internal year average dose** | mg/kg bw/day | 1.72E-07 | 2.09E-05 | 6.69E-05 |
| **Results: Integrated** | | | | |
| **Internal event dose** | mg/kg bw | 3.49E-06 | 3.49E-06 | 1.01E-01 |
| **Internal dose on day of exposure** | mg/kg bw/day | 3.49E-06 | 2.09E-05 | 1.01E-01 |
| **Internal year average dose** | mg/kg bw/day | 1.72E-07 | 2.09E-05 | 8.27E-04 |
| **Results: Mixing & Loading + Application** | | | | |
| **Results: Inhalation** | | | | |
| **Mean event concentration** | mg/m^3^ | 1.70E+01 | 1.70E+01 |  |
| **Peak concentration (TWA 15 min)** | mg/m^3^ | 1.83E+01 | 1.83E+01 |  |
| **Mean concentration on day of exposure** | mg/m^3^ | 3.53E-01 | 3.53E-01 |  |
| **Year average concentration** | mg/m^3^ | 2.91E-03 | 2.91E-03 |  |
| **External event dose** | mg/kg bw | 1.85E-01 | 1.85E-01 |  |
| **External dose on day of exposure** | mg/kg bw | 1.85E-01 | 1.85E-01 |  |
| **Internal event dose** | mg/kg bw | 9.25E-02 | 9.25E-02 |  |
| **Internal dose on day of exposure** | mg/kg bw/day | 9.25E-02 | 9.25E-02 |  |
| **Internal year average dose** | mg/kg bw/day | 7.60E-04 | 7.60E-04 |  |
| **Results: Dermal** | | | | |
| **Dermal load** | mg/cm^2^ | 6.23E-04 | 6.23E-04 |  |
| **External event dose** | mg/kg bw | 8.14E-03 | 8.14E-03 |  |
| **External dose on day of exposure** | mg/kg bw | 8.14E-03 | 8.16E-03 |  |
| **Internal event dose** | mg/kg bw | 8.14E-03 | 8.14E-03 |  |
| **Internal dose on day of exposure** | mg/kg bw/day | 8.14E-03 | 8.16E-03 |  |
| **Internal year average dose** | mg/kg bw/day | 6.71E-05 | 8.78E-05 |  |
| **Results: Integrated** | | | | |
| **Internal event dose** | mg/kg bw | 1.01E-01 | 1.01E-01 |  |
| **Internal dose on day of exposure** | mg/kg bw/day | 1.01E-01 | 1.01E-01 |  |
| **Internal year average dose** | mg/kg bw/day | 8.27E-04 | 8.48E-04 |  |

**Supplemental Table S10 – Input and Outputs for Primer in CEM**

| **Input** | **Units** | **Primer -Adult** |
| --- | --- | --- |
| **Product/Article** |  | Water-based wall paint |
| **Use Environment** |  | Residence- living room |
| **Models Selected** |  | Emission from Product applied to surface indoors, dermal dose from product applied to skin, fraction and permeability models |
| **Article Users and Activity Pattern** |  | Adult and SAH |
| **Use a User Defined Emission Rate?** |  | Let CEM define emission rate |
| **Dermal absorption or permeability?** |  | Permeability |
| **Use Near Field Zone?** |  | Do Not Use Near Field Zone 1 |
| **Inputs - Scenario** | | |
| **Background Air Concentration** | mg/m^3^ | 0 |
| **Weight fraction of chemical** |  | 6.00E-06 |
| **Inputs - Chemical Properties** | | |
| **Log Octanol water partition coeff** | 10log | 7.30E-01 |
| **Molecular weight** | g/mol | 8.61E+01 |
| **Saturation Conc in Air** | mg/m^3^ | 5.34E+05 |
| **Vapor pressure** | torr | 1.16E+02 |
| **Inputs - Product/Article Properties** |  |  |
| **Density of product** | g/cm^3^ | 1.25 |
| **Duration of use - acute** | min | 5.40E+02 |
| **Duration of use - chronic** | min | 2.40E+02 |
| **Frequency of use - acute** | events/day | 1 |
| **Frequency of use - chronic** | events/year | 7.00E+00 |
| **Mass of product used - acute** | g/use | 4.73E+03 |
| **Mass of product used - chronic** | g/use | 4.73E+03 |
| **Product Dilution Fraction** |  | 1.00E+00 |
| **Skin Permeability Coeff** | cm/hr | 2.31E-03 |
| **Inputs - Environmental Inputs** | | |
| **ACH, Zone 1** | per hour | 4.50E-01 |
| **ACH, Zone 2** | per hour | 4.50E-01 |
| **Building Volume** | m^3^ | 4.92E+02 |
| **Interzone Vent Rate** | m^3^/hr | 8.86E+01 |
| **Use Environment Volume** | m^3^ | 5.00E+01 |
|  |  |  |
| **Results: Inhalation** | | |
| **Acute Dose Rate** | mg/kg/d | 5.17E-05 |
| **Chronic Avg Daily Dose** | mg/kg/d | 1.30E-06 |
| **Chronic Dose** | mg/day | 5.42E-03 |
| **Chronic ADD** | mg/kg/d | 1.30E-06 |
| **Chronic ADC Zone 1** | mg/m^3^ | 5.77E-06 |
| **Chronic ADC Zone 2** | mg/m^3^ | 2.24E-07 |
| **Peak Dose** | mg/day | 4.13E-03 |
| **Peak Conc** | mg/m^3^ | 9.36E-04 |
| **Results: Dermal** | | |
| **Acute Dose Rate** | mg/kg/d | 2.45E-03 |
| **Chronic Avg Daily Dose** | mg/kg/d | 2.09E-05 |
| **Results: Total** | | |
| **Acute Dose Rate** | mg/kg/d | 2.50E-03 |
| **Chronic Avg Daily Dose** | mg/kg/d | 2.22E-05 |

**Supplemental Table S11 – Input and Outputs for Tablet Cover in CEM**

| **Input** | **Units** | **Tablet Cover - Child** |
| --- | --- | --- |
| **Product/Article** |  | Plastic articles: other objects with potential for routine contact (toys, foam blocks, tents) |
| **Use Environment** |  | Residence - Kitchen |
| **Models Selected** |  | Emission from Article, Ingestion after Inhalation, Ingestion of Article Mouthed, Incidental Dust Ingestion |
| **Article Users and Activity Pattern** |  | Child and SAH |
| **Inputs - Scenario** | | |
| **Background Air Concentration** | mg/m^3^ | 0 |
| **Background Dust Concentration** | ug/mg | 0 |
| **Initial Conc of SVOC in article** | mg/cm^3^ | 1.00E-05 |
| **Weight fraction of chemical** |  | 1.00E-07 |
| **Inputs - Chemical Properties** | | |
| **Gas Phase Mass Transfer Coeff** | m/hr | 1.60E+00 |
| **Henry's Law Coeff** | atm/M | 4.81E-04 |
| **Log Octanol-air Partition Coefficient** |  | 2.44E+00 |
| **Log Octanol water partition coeff** | 10log | 7.30E-01 |
| **Molecular weight** | g/mol | 8.61E+01 |
| **Overall Mass transfer coeff, abrased particle** | m/hr | 6.60E-03 |
| **Overall Mass transfer coeff, dust** | m/hr | 1.84E-02 |
| **overall mass transfer coeff, interior surface** | m/hr | 5.68E-03 |
| **overall mass transfer coeff, RP** | m/hr | 1.73 |
| **Saturation Conc in Air** | mg/m^3^ | 5.34E+05 |
| **Solid-Air Partition Coeff** |  | 1.69E+02 |
| **Solid-Phase Diffusion Coeff** | m^2^/hr | 8.41E-08 |
| **SVOC Partition Coeff, abrased particle** | m^3^/mg | 2.75E-08 |
| **SVOC Partition Coefficient, dust** | m^3^/mg | 2.75E-08 |
| **SVOC Partition Coeff, RP** | m^3^/mg | 1.10E-07 |
| **Vapor pressure** | torr | 1.16E+02 |
| **Water solubility** | mg/mL | 2.00E+01 |
| **Inputs - Article Properties** | | |
| **Absorption Fraction, Acute** |  | 1 |
| **Absorption Fraction, Chronic** |  | 1 |
| **Adherence Factor** | mg/cm^2^*event | 1 |
| **Area of Article Mouthed** | cm^2^ | 1.00E+01 |
| **Avg Molecule Diffusion per contact** | m | 7.10E-02 |
| **Chemical Migration Rate** | mg/cm^2^/hr | 1.00E-04 |
| **Density of Product/Article** | g/cm^3^ | 1.00E-01 |
| **Duration of Article Contact** | Min | 1.80E+02 |
| **Frequency of Article Contact** | events/day | 1 |
| **Ingestion Fraction - Dust** |  | 1.00E-01 |
| **Ingestion Fraction RP** |  | 1.00E-01 |
| **Ingestion Fraction, abraded particle** |  | 1.00E-01 |
| **Skim Permeability Coeff** | cm/hr | 2.31E-03 |
| **Surface Area of Article** | m^2^ | 6.70E-02 |
| **Thickness of Article Surface Layer** | M | 1.00E-01 |
| **Transdermal Permeability Coeff** | m/hr | 3.61E-05 |
| **Inputs - Environmental Inputs** |  |  |
| **Abraded Particle Deposition Rate Constant** | 1/hr | 2.34 |
| **Abraded Particle Resuspension Rate** | 1/hr | 1.29E-04 |
| **Ambient RP Conc** | mg/m^3^ | 5.20E-02 |
| **Area of Interior Surface** | m^2^ | 4.99E+01 |
| **Cleaning Efficiency** |  | 4.60E-01 |
| **Cleaning Frequency** | per hour | 6.00E-03 |
| **Density of Particle, dust** | mg/m^3^ | 2.00E+09 |
| **Density of Particle, RP** | mg/m^3^ | 1.00E+09 |
| **Deposition rate, dust** | per hour | 3.3 |
| **Deposition rate, RP** | per hour | 1 |
| **Generation Rate of abraded particles** | mg/hr | 5.31E-03 |
| **HVAC Filter Penetration for RP** |  | 5.00E-02 |
| **Interzone Vent Rate** | m^3^/hr | 8.86E+01 |
| **Mass Gen Rate, floor dust** | mg/hr | 2.53E+01 |
| **Mass Gen Rate, floor RP** | mg/hr | 0 |
| **Mass Gen Rate, suspended dust** | mg/hr | 1.18E+02 |
| **Mass Gen Rate, suspended rp** | mg/hr | 1.47E+01 |
| **Radius of abraded particle** | m | 7.00E-05 |
| **Radius of particle, dust** | m | 5.00E-04 |
| **Radius of particle, RP** | m | 5.00E-06 |
| **Resuspension rate, dust** | per day | 2.10E-04 |
| **Resuspension rate, RP** | per day | 2.60E-05 |
| **Thickness of Interior Surface** | m | 5.00E-03 |
| **Use Environment Volume** | m^3^ | 2.40E+01 |
| **Results: Inhalation** | | |
| **Chronic Steady-State Air SVOC** | μg/m^3^ | 9.41E+00 |
| **Chronic Gas Phase Chromic ADD** | mg/kg/d | 4.23E-03 |
| **Chronic Steady-State RP SVOC Conc** | μg/mg | 4.96E-03 |
| **Chronic Particulate Phase Chronic ADD** | mg/kg/d | 7.56E-06 |
| **Inhalation Total Chronic ADD** | mg/kg/d | 4.24E-03 |
| **Gas Phase ADR** | mg/kg/d | 4.26E-03 |
| **Particulate Phase ADR** | mg/kg/d | 7.64E-06 |
| **Inhalation Total ADR** | mg/kg/d | 4.27E-03 |
| **Results: Hand-to-Mouth** | | |
| **Background dust chronic ADD** | mg/kg/d | 0.00E+00 |
| **Mouthing Chronic ADD** | mg/kg/d | 1.81E-04 |
| **Ingestion after Inhalation Chronic ADD** | mg/kg/d | 1.06E-06 |
| **Incidental Dust Chronic ADD** | mg/kg/d | 3.51E-04 |
| **Max Incidental Dust Chronic ADD** | mg/kg/d | 3.51E+05 |
| **Mouthing ADR** | mg/kg/d | 1.81E-04 |
| **Acute- Ingestion after Inhalation ADR** | mg/kg/d | 1.06E-06 |
| **Acute- Incidental Dust ADR** | mg/kg/d | 3.78E-04 |
| **Max Incidental Dust ADR** | mg/kg/d | 3.78E+05 |
| **Results: Dermal** | | |
| **Skin Contact Chronic ADD** | mg/kg/d | 1.95E-05 |
| **Vapor to Skin Chronic ADD** | mg/kg/d | 1.85E-06 |
| **Total Chronic ADD** | mg/kg/d | 2.18E-03 |
| **Skin Contact ADR** | mg/kg/d | 1.95E-05 |
| **Vapor to Skin ADR** | mg/kg/d | 1.87E-06 |
| **Total ADR** | mg/kg/d | 2.34E-03 |
| **Results: Total** | | |
| **Acute Dose Rate** | mg/kg/d | 7.17E-03 |
| **Chronic Avg Daily Dose** | mg/kg/d | 6.95E-03 |

**Supplemental Table S12 – Input and Outputs for Shelf Liner in CEM**

| **Input** | **Units** | **Shelf Liner - Adult** |
| --- | --- | --- |
| **Product/Article** |  | Plastic articles: Vinyl Flooring |
| **Use Environment** |  | Residence- Kitchen |
| **Models Selected** |  | Emission from Article, Calculation of Inhalation Dose, Ingestion after Inhalation, Ingestion of Article Mouthed, Incidental Dust Ingestion |
| **Article Users and Activity Pattern** |  | Adult and SAH |
| **Inputs - Scenario** | | |
| **Background Air Concentration** | mg/m^3^ | 0 |
| **Background Dust Concentration** | μg/mg | 0 |
| **Initial Conc of SVOC in article** | mg/cm^3^ | 1.00E-05 |
| **Weight fraction of chemical** |  | 1.00E-07 |
| **Inputs - Chemical Properties** | | |
| **Gas Phase Mass Transfer Coeff** | m/hr | 3.23E+00 |
| **Henry's Law Coeff** | atm/M | 4.81E-04 |
| **Log Octanol-air Partition Coefficient** |  | 2.44E+00 |
| **Log Octanol water partition coeff** | 10log | 7.30E-01 |
| **Molecular weight** | g/mol | 8.61E+01 |
| **Overall Mass transfer coeff, abrased particle** | m/hr | 6.60E-03 |
| **Overall Mass transfer coeff, dust** | m/hr | 1.84E-02 |
| **Overall Mass transfer coeff, interior surface** | m/hr | 5.68E-03 |
| **Overall Mass transfer coeff, RP** | m/hr | 1.73E+00 |
| **Saturation Conc in Air** | mg/m^3^ | 5.34E+05 |
| **Solid-Air Partition Coeff** |  | 1.69E+02 |
| **Solid-Phase Diffusion Coeff** | m^2^/hr | 8.41E-08 |
| **SVOC Partition Coeff, abrased particle** | m^3^/mg | 2.75E-08 |
| **SVOC Partition Coeff, RP** | m^3^/mg | 1.10E-07 |
| **Vapor pressure** | torr | 1.16E+02 |
| **Water solubility** | mg/mL | 2.00E+01 |
| **Inputs - Article Properties** | | |
| **Absorption Fraction, Acute** |  | 1 |
| **Absorption Fraction, Chronic** |  | 1 |
| **Adherence Factor** | mg/cm^2^*event | 1 |
| **Area of Article Mouthed** | cm^2^ | 0 |
| **Avg Molecule Diffusion per contact** | m | 2.90E-02 |
| **Chemical Migration Rate** | mg/cm^2^/hr | 1.00E-04 |
| **Density of Product/Article** | g/cm^3^ | 1.00E-01 |
| **Duration of Article Contact** | Min | 3.00E+01 |
| **Frequency of Article Contact** | events/day | 5 |
| **Ingestion Fraction - Dust** |  | 1.00E-01 |
| **Ingestion Fraction TSP** |  | 1.00E-01 |
| **Ingestion Fraction, abraded particle** |  | 1.00E-01 |
| **Skim Permeability Coeff** | cm/hr | 2.31E-03 |
| **Surface Area of Article** | m^2^ | 3.6 |
| **Thickness of Article Surface Layer** | m | 1.00E-01 |
| **Transdermal Permeability Coeff** | m/hr | 3.61E-05 |
| **Inputs - Environmental Inputs** |  |  |
| **Abraded Particle Deposition Rate Constant** | 1/hr | 2.34 |
| **Abraded Particle Resuspension Rate** | 1/hr | 1.29E-04 |
| **Ambient RP Conc** | mg/m^3^ | 5.20E-02 |
| **Area of Interior Surface** | m^2^ | 4.99E+01 |
| **Cleaning Efficiency** |  | 4.60E-01 |
| **Cleaning Frequency** | per hour | 6.00E-03 |
| **Density of Particle, dust** | mg/m^3^ | 2.00E+09 |
| **Density of Particle, RP** | mg/m^3^ | 1.00E+09 |
| **Deposition rate, dust** | per hour | 3.3 |
| **Deposition rate, RP** | per hour | 1 |
| **Generation Rate of abraded particles** | mg/hr | 5.31E-03 |
| **HVAC Filter Penetration for RP** |  | 5.00E-02 |
| **Interzone Vent Rate** | m^3^/hr | 8.86E+01 |
| **Mass Gen Rate, floor dust** | mg/hr | 2.53E+01 |
| **Mass Gen Rate, floor RP** | mg/hr | 0 |
| **Mass Gen Rate, suspended dust** | mg/hr | 1.18E+02 |
| **Mass Gen Rate, suspended rp** | mg/hr | 1.47E+01 |
| **Radius of abraded particle** | m | 7.00E-05 |
| **Radius of particle, dust** | m | 5.00E-04 |
| **Radius of particle, RP** | m | 5.00E-06 |
| **Resuspension rate, dust** | per day | 2.10E-04 |
| **Resuspension rate, RP** | per day | 2.60E-05 |
| **Thickness of Interior Surface** | m | 5.00E-03 |
| **Use Environment Volume** | m^3^ | 2.40E+01 |
| **Results: Inhalation** | | |
| **Acute Dose Rate** | mg/kg/d | 1.44E-03 |
| **Chronic Avg Daily Dose** | mg/kg/d | 1.43E-03 |
| **Chronic Steady-State Air SVOC** | μg/m^3^ | 9.41E+00 |
| **Chronic Gas Phase Chromic ADD** | mg/kg/d | 1.43E-03 |
| **Chronic Steady-State RP SVOC Conc** | μg/mg | 4.96E-03 |
| **Chronic Particulate Phase Chronic ADD** | mg/kg/d | 2.55E-06 |
| **Result: Hand-to-Mouth** | | |
| **Acute Dose Rate** | mg/kg/d | 2.67E-05 |
| **Chronic Avg Daily Dose** | mg/kg/d | 2.48E-05 |
| **Ingestion after Inhalation Chronic ADD** | mg/kg/d | 3.57E-07 |
| **Incidental Dust Chronic ADD** | mg/kg/d | 2.45E-05 |
| **Max Incidental Dust Chronic ADD** | mg/kg/d | 2.45E+04 |
| **Acute- Ingestion after Inhalation ADR** | mg/kg/d | 3.57E-07 |
| **Acute- Incidental Dust ADR** | mg/kg/d | 2.63E-05 |
| **Max Incidental Dust ADR** | mg/kg/d | 2.63E+04 |
| **Results: Dermal** | | |
| **Acute Dose Rate** | mg/kg/d | 2.62E-03 |
| **Chronic Avg Daily Dose** | mg/kg/d | 2.44E-03 |
| **Skin Contact Chronic ADD** | mg/kg/d | 1.80E-06 |
| **Vapor to Skin Chronic ADD** | mg/kg/d | 4.19E-07 |
| **Total Chronic ADD** | mg/kg/d | 2.44E-03 |
| **Skin Contact ADR** | mg/kg/d | 1.80E-06 |
| **Vapor to Skin ADR** | mg/kg/d | 4.22E-07 |
| **Total ADR** | mg/kg/d | 2.62E-03 |
| **Results: Total** | | |
| **Acute Dose Rate** | mg/kg/d | 4.09E-03 |
| **Chronic Avg Daily Dose** | mg/kg/d | 3.89E-03 |

**Table S13 – Summary of VAM concentrations in consumer products, with estimated detection limits**

| **Category** | **Sample Description** | **ppmw VAM** | **%RSD** | **Comments** |
| --- | --- | --- | --- | --- |
| Caulks/Sealants | Latex Caulk* | ND <2 | NA |  |
|  | Multi-Purpose White Caulk | 7.6 | 6.1 |  |
|  | Painters Caulk | 2.3 | 9.4 |  |
|  | Tub and Tile Acrylic Caulk* | ND <0.4 | NA |  |
|  | Tub and Tile Sealant* | ND <0.4 | NA |  |
|  | White Vinyl Adhesive Caulk* | ND <0.5 | NA |  |
| Cosmetics/Personal Care | Eye Mask Pads* | ND <0.1 | NA |  |
|  | Eye Drop Solution* | ND <0.3 | NA |  |
|  | Facial Serum* | ND <0.3 | NA |  |
|  | Children’s Lip Gloss* | ND <0.2 | NA | Limited sample |
|  | Eye Pencil* | ND <0.6 | NA | Very limited sample |
|  | Eyeliner Pen*` | ND <0.6 |  |  |
|  | Face Mask* | ND <0.2 | NA |  |
|  | Blackhead Remover Mask* | ND <0.1 | NA |  |
|  | Hot Pain Relief Patch* | ND <0.1 | NA |  |
| Glues | Tacky Glue | 1 | 2.9 |  |
|  | Wood Glue | 59 | 4.9 |  |
|  | Book Glue | 1.5 | 0.7 |  |
|  | Clear School Glue* | ND <0.1 | NA |  |
|  | Multi-Purpose Glue | 52 | 2.1 |  |
|  | Rubber Flooring Adhesive* | ND <0.7 | NA |  |
|  | Cove Base and Stair Tread Adhesive* | ND <2 | NA |  |
|  | Interior Projects Adhesive* | ND <1 | NA |  |
|  | Arts and Crafts Glue | 648 | 0.5 | One outlier, result >> than +200 mg std |
|  | Concrete Bonding Adhesive** | ND <0.4 | NA | LOD based upon +200 mg standard |
|  | Concrete Glue* | ND <0.2 | NA |  |
|  | Glue Stick**^ | NA | NA | VAM not detected at +200 mg level |
|  | All-Purpose White Glue | 44 | 7.2 | Lower precision in calibration (R^2^=0.990) |
|  | Premium Wood Glue | 49 | 3.2 |  |
|  | Seam and Repair Adhesive | 74 | 3.8 |  |
| Ingestibles | Glucosamine Tablets* | ND <0.1 | NA |  |
| Other | Slime Kit* | ND <0.2 | NA |  |
|  | Laundry Detergent Pods** | ND <36 | NA | VAM barely detected at +200 mg level |
| Paint | Latex Enamel* | ND <0.4 | NA |  |
|  | Acrylic Paint* | ND <0.4 | NA |  |
|  | Underlayment Primer** | ND <0.3 | NA | LOD based upon +200 mg standard |
|  | Garage Floor Coating**^ | NA | NA | VAM not detected at +200 mg level |
|  | Multi-Surface Interior Latex Primer** | ND <6 | NA | VAM barely detected at +200 mg level |
| Solid Article | Facial Sponge* | ND <0.1 | NA |  |
|  | Memory Foam Slippers* | ND <0.1 | NA |  |
|  | Macrame Board* | ND <0.1 | NA |  |
|  | Plastic Shelf Liner* | ND <0.1 | NA |  |
|  | Sponge Mop Refill* | ND <0.1 | NA |  |
|  | Non-Slip Refrigerator Mats* | ND <0.1 | NA |  |
|  | Golf Shoes* | ND <0.1 | NA |  |
|  | Shower Curtain Liner* | ND <0.1 | NA |  |
|  | Industrial Ducting Hose* | ND <0.1 | NA |  |
|  | Protective Knee Pads* | ND <0.3 | NA | Very low-density foam |
|  | Optic Fiber Splice Sleeves* | ND <0.1 | NA |  |
|  | Soft Foam Rainbow Play Balls* | ND <0.1 | NA |  |
|  | Kids Protective Case Cover for Tablet* | ND <0.1 | NA |  |
|  | Foam Embroidery Sheets* | ND <0.2 | NA | Low density foam |
|  | Interlocking Foam Portable Flooring* | ND <0.1 | NA | Low density foam |
|  | Pencil Case* | ND <0.1 | NA |  |
| Surface Treatment | Waterproofing Kit - Liquid* | ND <0.3 | NA |  |
|  | Waterproofing Kit - Powder**^ | NA | NA | VAM not detected at +200 mg level |
|  | Thin Set Mortar** | ND <7 | NA | VAM barely detected at +200 mg level |
|  | Spackle**^ | NA | NA | VAM not detected at +200 mg level |
|  | Latex Plastic Wood Filler** | ND <16 | NA | VAM barely detected at +200 mg level |
|  | Lightweight Joint Compound** | ND <1600 | NA | VAM barely detected at +200 mg level |
|  | PVA Release Agent** | ND <0.2 | NA | LOD based upon +200 mg standard |
|  | Universal Underlayment**^ | NA | NA | VAM not detected at +200 mg level |
|  | Concrete Resurfacer (Dry)** | *16* | 3.5 | Estimated, nonlinear calibration |
|  | Concrete Bonding Agent | 22 | 3.4 |  |
|  | Liquid Easy Casting Mold Release* | ND <0.2 | NA |  |
|  | Spackle**^ | NA | NA | VAM not detected at +200 mg level |
|  | Self-Leveling Underlayment** | ND <6 | NA | VAM barely detected at +200 mg level |
|  | Spackling Paste** | ND <114 | NA | VAM barely detected at +200 mg level |
|  | Cover Coat Compound** | ND <13 | NA | VAM barely detected at +200 mg level |
|  | Topping Joint Compound** | ND <180 | NA | VAM barely detected at +200 mg level |
|  | Wallboard Joint Compound** | ND <0.7 | NA | LOD based upon +200 mg standard |

ND: not detected

NA: not applicable
* VAM not detected; sample exhibits typical calibration behavior and detection limits

**Samples exhibits atypical calibration behavior and detection limits

^ Samples exhibiting unusual partitioning and/or calibration behavior

**References for Supplementary Tables:**

1. Lide DR (ed). CRC Handbook of Chemistry and Physics. 76th ed. Boca Raton, FL: CRC Press Inc., 1995-1996. pp. 3–7.
2. Budavari S (ed). The Merck Index - An Encyclopedia of Chemicals, Drugs, and Biologicals. Whitehouse Station, NJ: Merck and Co., Inc., 1996. p. 1705.
3. Ullmann Encyclopedia of Industrial Chemistry. 1995. Volume A22; A27.
4. Riddick JA, Bunger WB. Organic Solvents: Physical Properties and Methods of Purification. Techniques of chemistry. 4th ed. Wiley-Interscience, 1986. pp. 1325.
5. Haynes WM (ed). CRC Handbook of Chemistry and Physics. 97^th^ ed. Boca Raton, FL: CRC Press, 2016.
6. Verschueren K. Handbook of Environmental Data on Organic Chemicals. 2nd ed. New York, NY: Van Nostrand Reinhold Co., 1986. 633, 1184–1185.
7. [PCKOCWIN] Organic Carbon Partition Coefficient Program for Windows [Estimation Model]. 2000. Version 1.66. Washington (DC); Syracuse (NY): US Environmental Protection Agency, Office of Pollution Prevention and Toxics; Syracuse Research Corporation.
8. Hazardous Substance Databank (HSDB). Hazardous Substances Data Bank. 1991. National Library of Medicine, National Toxicology Information Program, Bethesda, MD.
